# Phylogenetic Analysis of *Klebsiella pneumoniae* from Hospitalized Children, Pakistan

**DOI:** 10.3201/eid2311.170833

**Published:** 2017-11

**Authors:** Hasan Ejaz, Nancy Wang, Jonathan J. Wilksch, Andrew J. Page, Hanwei Cao, Shruti Gujaran, Jacqueline A. Keane, Trevor Lithgow, Ikram ul-Haq, Gordon Dougan, Richard A. Strugnell, Eva Heinz

**Affiliations:** CAMS, Aljouf University, Aljouf, Saudi Arabia; The Children’s Hospital, Lahore, Pakistan (H. Ejaz);; The University of Melbourne, Melbourne, Victoria, Australia (H. Ejaz, N. Wang, J.J. Wilksch, H. Cao, S. Gujaran, R.A. Strugnell);; Wellcome Trust Sanger Institute, Hinxton, UK (A.J. Page, J.A. Keane, G. Dougan, E. Heinz);; Monash University, Melbourne (T. Lithgow, E. Heinz); Government College University, Lahore (I.ul-Haq)

**Keywords:** *Klebsiella*, ESBL, extended-spectrum β-lactamase, antimicrobial resistance, neonatal infection, multidrug resistance, Pakistan, bacteria, Australia, United Kingdom, Klebsiella pneumonia, children

## Abstract

*Klebsiella pneumoniae* shows increasing emergence of multidrug-resistant lineages, including strains resistant to all available antimicrobial drugs. We conducted whole-genome sequencing of 178 highly drug-resistant isolates from a tertiary hospital in Lahore, Pakistan. Phylogenetic analyses to place these isolates into global context demonstrate the expansion of multiple independent lineages, including *K. quasipneumoniae*.

*Klebsiella* spp. are gram-negative bacteria that are widely distributed in the environment, and *K. pneumoniae* is a common cause of infection in humans (*1*). Increasingly, *K. pneumoniae* is reported as a cause of invasive bloodborne infections, particularly in healthcare settings and in immunocompromised patients (*2*). Of concern is that infection-associated *K. pneumoniae* is often multidrug resistant (MDR) and can harbor resistance determinants against most, if not all, commonly used antimicrobial drugs, posing a major threat to public health. The World Health Organization recently highlighted finding new treatments against MDR *Enterobacteriaceae* (including *Klebsiella*) as priority 1 (critical) (http://www.who.int/mediacentre/news/releases/2017/bacteria-antibiotics-needed/en/).

*K. pneumoniae* is a major pathogen in economically developed settings, and multiple outbreaks in different countries have been reported. Less is known about its prevalence in economically challenged areas, including lower and middle income countries (LMIC). Reports are now appearing about *Klebsiella*-associated infections in Nepal (*3*) and in Indonesia, Laos, and Vietnam (*1*). *Klebsiella* can spread rapidly in hospital environments, and the increasing prevalence of MDR strains has raised concern among major health organizations (*4*,^5^). Thus, high-resolution insight into the diversity of *Klebsiella* spp. isolated in LMICs will provide vital data for improving epidemiologic management of infections and for better understanding of the mechanisms of spread between LMICs and more developed countries.

## The Study

**Figure 1:**
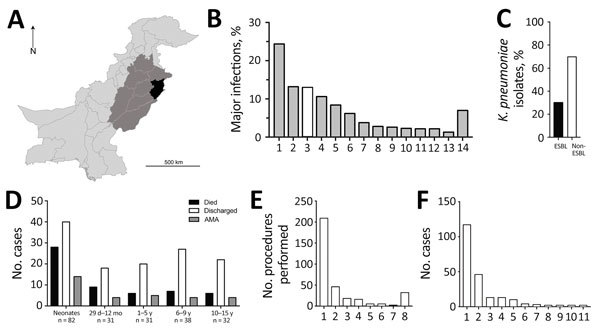
Statistical overview of bacterial isolates from clinical samples collected during May 2010–February 2012 from The Children’s Hospital & The Institute of Child Health, Lahore, Pakistan. A) Map of Pakistan highlighting the main catchment area of Lahore (black, population ≈10 million) and the wider area of Punjab (medium gray, population ≈100 million). B) A total of 5,475 samples collected from children resulted in laboratory-positive cultures; the 5 most frequently occurring bacterial species accounted for ≈70% of total bacterial infections, and *Klebsiella pneumoniae* (white bar) was the third most dominant (710 isolates). 1, *Escherichia coli*; 2, coagulase-negative *Staphylococcus*; 3, *K. pneumoniae*; 4, *Pseudomonas aeruginosa*; 5, *K. oxytoca*; 6, *Staphylococcus aureus*; 7, *Acinetobacter* spp.; 8, *Enterococcus faecalis*; 9, *Citrobacter* spp.; 10, *Streptococcus pyogenes*; 11, *Burkhoderia cepacia*; 12, *Enterobacter clocae*; 13, *Salmonella enterica* var. Typhi; 14, others (>100 species). C) The proportion of ESBL-producing *K. pneumoniae* (214 isolates) among all *K. pneumoniae* isolates demonstrated high prevalence of antimicrobial resistance. D) A total of 38.3% of ESBL-producing *K. pneumoniae* infections occurred in neonates (<29 d), an age group that also showed the highest fatality rate (34.1%). Patients who were removed from the hospital against medical advice (AMA) typically were critically ill and were taken home by the family to avoid dying in the hospital. E) The apparent hierarchy shown in panel E closely correlated with interventions given. IV line (97.7%), urinary catheter (27.5%), and ETT (8.4%) were the 3 most commonly administered procedures among sampled patients, although no temporal relationship between procedure and sample collection could be established. 1, IV line; 2, urinary catheter; 3, ETT; 4, PD catheter; 5, surgery; 6, NG tube; 7, CVP; 8, others. F) A total of 54.6% of ESBL-producing *K. pneumoniae* isolates were from patient blood samples, followed by urine (21.5%), CSF (6%), and ETT (6%). 1, Blood; 2, urine; 3, CSF; 4, ETT; 5, PD catheter; 6, tracheal secretions; 7, pus; 8, CVP tip; 9, ear swab; 10, pleural fluid; 11, wound swab. CSF, cerebrospinal fluid; CVP, central venous catheter tip; ESBL, extended-spectrum β-lactamase; ETT, endotracheal tube; IV, intravenous; NG, nasogastric; PD, peritoneal dialysis catheter. The regional map was derived from the Global Administrative Areas online resource (http://www.gadm.org/).

Clinical samples were collected during a 22-month period (May 2010–February 2012) from The Children’s Hospital & The Institute of Child Health, Lahore (Lahore, Pakistan), the largest tertiary care hospital in the region (Figure 1, panel A). The hospital had a capacity of 650 beds during the study period but is under pressure to handle up to 2,000 inpatients at any given time. The primary catchment area is Lahore (population ≈10 million); the hospital also receives patients from the greater area of Punjab province (population ≈100 million) (Figure 1, panel A). The Ethical Committee of The Children’s Hospital & Institute of Child Health, Lahore, approved the study.

A total of 44,260 samples were collected in the course of routine sampling from children; 5,475 (12.4%) resulted in laboratory-positive cultures. Of these, 710 (13.0%) samples were positively identified as *K. pneumoniae*, the third most dominant isolate after *Escherichia coli* (1,336 [24.4%]) and coagulase-negative staphylococci (724 [13.2%]) (Figure 1, panel B). We screened all *K. pneumoniae* isolates for resistance to ceftazidime (30 μg disc, zone of inhibition <17 mm) or cefotaxime (30 μg disc, zone of inhibition <22 mm). We further tested *K. pneumoniae* isolates that were resistant to any of these indicator drugs using the Clinical and Laboratory Standards Institute combined-disc confirmatory test (*6*); extended-spectrum β-lactamase (ESBL) production was confirmed when the zone of inhibition by either cephalosporin drug increased by >5 mm in the presence of clavulanate. A total of 214 of *K. pneumoniae* isolates were ESBL-positive (Figure 1, panel C); most were isolated from children with bloodstream infections (Figure 1, panel D). The outcomes were severe, especially among neonatal patients (Figure 1, panel D); 56 died, 31 were taken home against medical advice, and 127 were discharged (Figure 1, panel D). Almost all patients infected with ESBL *Klebsiella* had received an intravenous line (209 [97.7%]) (Figure 1, panel E,F), and a high number received a urinary catheter (46 [21.5%]).

**Figure 2:**
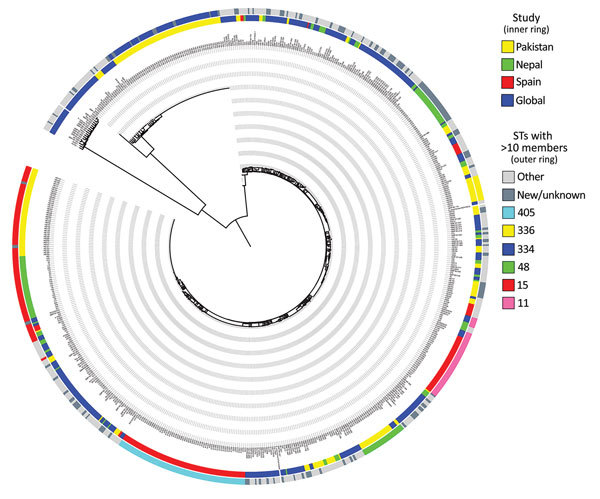
Phylogenetic analysis demonstrating the diversity of *Klebsiella pneumoniae* isolates from clinical samples collected during May 2010–February 2012 from The Children’s Hospital & The Institute of Child Health, Lahore, Pakistan, in a global context. The core gene tree based on the alignment derived from Roary (*12*) was calculated using RAxML (*14*) and shows the wide diversity of samples analyzed in this study (inner ring, yellow) in context with a large-scale global analysis (inner ring, blue [*4*]) and 2 hospital outbreaks, which show a more clonal pattern (inner ring: red, outbreak in Spain [*11*]; green, outbreak in Nepal [*3*]). The sequence types observed (outer ring) also reflect the diversity; most sequence types have <10 members even in this combined collection. STs, sequence types.

We performed whole-genome sequencing on 178 isolates (Technical Appendix Table 1). We prepared Illumina sequencing libraries (Illumina, San Diego, CA, USA) with a 450-bp insert size according to the manufacturer’s protocols and sequenced them on an Illumina HiSeq2000 with 100-bp–long paired-end reads before assembly using an open-source high-throughput assembly and improvement pipeline as described (*7*) (https://github.com/sanger-pathogens/) and annotated using prokka (*8*). Initial clustering using mash (*9*) enabled aligning of these isolates to published reference sequences (Technical Appendix Figure 1, panel A). The clustering indicated a strong structure for the isolates that fell within the species *K. pneumoniae* (Technical Appendix Figure 1, panel B). However, the analysis also revealed a large group of sequences most similar to *K. quasipneumoniae*; closer inspection focusing on this species showed strongest similarity to subspecies *similipneumoniae* (Technical Appendix Figure 1, panel C) (*10*). We combined several independent datasets: a large global collection (*1*); 2 hospital outbreaks obtained in a comparable time frame, 1 of which was based in Nepal in 2012 (*3*); and a hospital study from Spain that also focused on diversity within ESBL-producing strains (*11*) (Technical Appendix Table 2). We applied the pan-genome pipeline Roary version 3.7.0 (*12*) with a blastp (https://blast.ncbi.nlm.nih.gov/Blast.cgi?PAGE=Proteins) percentage identity of 90% and a core definition of 99%, resulting in a core gene alignment comprising 1,793 genes for all studies (Figure 2) and 3,486 genes for the strains of this study (Technical Appendix Figure 2). We first extracted single-nucleotide polymorphisms using snp-sites version 2.3.2 (*13*), then calculated a maximum-likelihood tree using RAxML version 8.2.8 (*14*) with the general time-reversible model and 100 bootstrap repeats. The core gene phylogeny (Figure 2) shows a wide distribution of the isolates from Pakistan across different lineages rather than 1 clonal lineage. The diversity of our strain collection is further emphasized through the diversity of multilocus sequence types (STs). No single ST dominates (Figure 2 outer ring; Technical Appendix Figure 2); however, a large group of isolates belongs to ST15, which is known to be problematic. The presence of *K. quasipneumoniae* isolates agrees with an overall lower percentage of reads mapped against *K. pneumoniae* (Technical Appendix Table 1) and with recent descriptions of virulent *K. quasipneumoniae* strains (*1*,*9*,*15*). Assessing the metadata in phylogenetic context highlights the association of the *K. quasipneumoniae* lineage with patients in the neonatal ward, suggestive of its nosocomial residency (Technical Appendix Figure 2). However, other main lineages (e.g., ST15, ST48) show a dynamic spread across wards and age groups, indicating against >1 resident lineages but instead a frequent movement of *K. pneumoniae* through the hospital, general population, or both.

The high number of *K. quasipneumoniae* isolates, even if potentially restricted to most sequences derived from a lineage potentially resident in a specific ward, highlights the importance of a diverse set of sampling sites to be studied. It also highlights the need for continued monitoring of new emerging strains and that our knowledge of the diversity of potentially problematic lineages is far from exhaustive.

## Conclusions

The *Klebsiella* isolates in this study represented the *Klebsiella* isolates routinely present in infections over a protracted period. Our findings highlight a consistent problem with ESBL-encoding strains belonging to a multitude of lineages. We observed sporadic single-isolate lineages, as well as smaller, related clusters of 5–10 strains per lineage, in addition to 2 larger clusters of strains. More studies are needed to better delineate the distinguishing features for successful spread and persistence of lineages such as the ST15 cluster. Also, the large spread of *K. quasipneumoniae* is unusual. Further intense monitoring of LMIC hospital environments is urgently needed to prevent the persistence of resident lineages with very high base-level drug resistance, which, through the inevitable acquisition of a few more genes, would lead to untreatable infections.

Technical Appendix Details of sequenced *Klebsiella pneumoniae* strains from children, Pakistan; GenBank accession numbers of published *K. pneumoniae* strains included in this analysis; whole-genome clustering; patient metadata in phylogenetic context.
