# Supplementary material for: Phylogenetic Analysis of Klebsiella pneumoniae from Hospitalized Children, Pakistan
Source: Emerg Infect Dis. 2017 Nov;23(11):1872–5. doi: 10.3201/eid2311.170833 (PMC5652443; doi:10.3201/eid2311.170833)
Supplement: Technical Appendix — Details of sequenced Klebsiella pneumoniae strains from children, Pakistan; GenBank accession numbers of published K. pneumoniae strains included in this analysis; whole-genome clustering; patient metadata in phylogenetic context. [file 17-0833-Techapp-s1.pdf]

Article DOI: https://doi.org/10.3201/eid2311.170833

Phylogenetic Analysis of *Klebsiella pneumoniae* from Hospitalized Children, Pakistan

Technical Appendix

Technical Appendix Table 1. Details of sequenced *Klebsiella pneumoniae* strains

Lane	Total yield (k	Matches to r	Total length	No contigs	Average contig length	Largest contig	N50	Contigs in N50	Total raw reads	Reads mapped	Accession	Strain	species	kraken % ma	Assembly accession	Sample accession	Scaffolds accessions
14893_8#69	550679	46	5906670	116	50919.57	768351	365337	6	5833546	5189300	ERR775485	HE001	quasipneum	54.62	GCA_900180695	ERS482876	FXMW01000001-FXMW01000116
14893_8#70	593330	60.4	5433377	30	181112.57	1446564	489677	3	6291674	6034838	ERR775486	HE002	pneumoniae	87.15	GCA_900180485	ERS482878	FXND01000001-FXND01000030
14893_8#71	555896	60.2	5436516	40	135912.9	1827693	1003766	2	5897468	5655206	ERR775487	HE003	pneumoniae	86.97	GCA_900180975	ERS482881	FXMI01000001-FXMI01000040
14893_8#72	571072	58.9	5475183	75	73002.44	1106476	726247	3	6062186	5802981	ERR775488	HE004	pneumoniae	85.48	GCA_900180545	ERS482884	FXMV01000001-FXMV01000075
14893_8#73	718763	60.1	5430710	24	226279.58	1446161	737727	3	7624760	7318761	ERR775489	HE005	pneumoniae	86.92	GCA_900180795	ERS482888	FXMA01000001-FXMA01000024
14893_8#74	555714	52.8	5857395	104	56321.11	412833	246022	10	5911852	5583350	ERR775490	HE006	pneumoniae	82.93	GCA_900180945	ERS482892	FXMF01000001-FXMF01000104
14893_8#75	574968	52.4	5836926	101	57791.35	703958	238428	9	6084302	5796883	ERR775491	HE007	pneumoniae	82.4	GCA_900180675	ERS482895	FXMG01000001-FXMG01000101
14893_8#76	585676	45	5873752	109	53887.63	1967752	1046625	2	6198202	5926958	ERR775492	HE008	quasipneum	50.77	GCA_900180495	ERS482899	FXNL01000001-FXNL01000109
14893_8#77	562799	59.5	5476568	76	72060.11	1624484	427376	4	5971278	5721244	ERR775493	HE009	pneumoniae	86.07	GCA_900180525	ERS482902	FXMU01000001-FXMU01000076
14893_8#81	722798	52.3	5890088	98	60102.94	621375	248385	9	7639992	7339474	ERR775495	HE013	pneumoniae	83.81	GCA_900180835	ERS482914	FXNC01000001-FXNC01000098
14893_8#82	656486	59.5	5432867	25	217314.68	1859094	1078469	2	7103828	6710905	ERR775496	HE014	pneumoniae	86.84	GCA_900180735	ERS482917	FXNW01000001-FXNW01000025
14893_8#83	604724	54.7	5839704	70	83424.34	698645	227886	9	6412564	6152077	ERR775497	HE015	pneumoniae	84.59	GCA_900180595	ERS482920	FXMC01000001-FXMC01000070
14893_8#84	598862	53.7	5854566	83	70536.94	406013	238609	10	6340970	5826250	ERR775498	HE016	pneumoniae	81.65	GCA_900180665	ERS482923	FXNE01000001-FXNE01000083
14893_8#87	622886	53.1	5832651	109	53510.56	468560	265092	9	6594968	6288183	ERR775501	HE019	pneumoniae	83.2	GCA_900180965	ERS482931	FXNB01000001-FXNB01000109
14893_8#88	599138	60.2	5432989	30	181099.63	1683753	1507719	2	6367256	6098049	ERR775502	HE020	pneumoniae	87.08	GCA_900180655	ERS482934	FXLZ01000001-FXLZ01000030
14893_8#89	702311	59.4	5639752	55	102540.95	1603009	1319118	2	7454146	7142119	ERR775503	HE021	pneumoniae	85.96	GCA_900180995	ERS482937	FXNY01000001-FXNY01000055
14893_8#90	1114544	56.4	5626570	79	71222.41	1991523	928911	2	11805170	11289189	ERR775504	HE022	pneumoniae	84.01	GCA_900181015	ERS482940	FXOD01000001-FXOD01000079
14893_8#91	658203	48.1	5577588	68	82023.35	1223653	484011	4	6983194	6658245	ERR775505	HE023	quasipneum	46.32	GCA_901181025	ERS482943	FXOA01000001-FXOA01000068
14893_8#92	626168	57.2	5621475	78	72070.19	1602571	427301	4	6660882	6342259	ERR775506	HE024	pneumoniae	84.66	GCA_900181075	ERS482946	FXOM01000001-FXOM01000078
14936_2#1	390669	57.2	5504096	50	110081.92	740075	414801	6	4292610	3906116	ERR775507	HE120	pneumoniae	84.44	GCA_900181035	ERS482939	FXOB01000001-FXOB01000050
14936_2#11	373207	55.6	5633718	83	67876.12	641872	359837	6	4089884	3717003	ERR775517	HE130	pneumoniae	83.25	GCA_900181315	ERS482967	FXPI01000001-FXPI01000083
14936_2#12	381261	52.5	5778120	77	75040.52	621528	222753	9	4175612	3812706	ERR775518	HE131	pneumoniae	82.4	GCA_900181275	ERS482969	FXOW01000001-FXOW01000077
14936_2#13	424937	54.6	5608003	79	70987.38	512648	348657	7	4654448	4194163	ERR775519	HE132	pneumoniae	84.92	GCA_900181295	ERS482971	FXPD01000001-FXPD01000079
14936_2#14	434713	51.9	5885123	128	45977.52	621562	281256	8	4748572	4332844	ERR775520	HE133	pneumoniae	81.9	GCA_900181305	ERS482973	FXPG01000001-FXPG01000128
14936_2#16	487943	50.4	5851179	87	67254.93	1150443	446532	5	5320584	4851456	ERR775522	HE135	pneumoniae	78.41	GCA_900181365	ERS482978	FXPF01000001-FXPF01000087
14936_2#17	385813	52.5	5893343	125	47146.74	621510	280819	8	4221462	3846354	ERR775523	HE136	pneumoniae	82.11	GCA_900181445	ERS482980	FXPQ01000001-FXPQ01000125
14936_2#19	395269	50	5885695	142	41448.56	621428	194087	10	4324814	3938245	ERR775525	HE138	pneumoniae	81.54	GCA_900181345	ERS482985	FXPL01000001-FXPL01000142
14936_2#2	411413	53.3	5619881	69	81447.55	621560	240372	8	4508334	4107263	ERR775508	HE121	pneumoniae	81.96	GCA_900181115	ERS482942	FXOH01000001-FXOH01000069
14936_2#20	427917	54.5	5625611	58	96993.29	621402	281540	8	4681916	4282008	ERR775526	HE139	pneumoniae	84.31	GCA_900181415	ERS482987	FXPS01000001-FXPS01000058
14936_2#21	440019	48.7	6079659	466	13046.48	654599	208918	10	4798626	4117813	ERR775527	HE140	pneumoniae	76.33	GCA_900181455	ERS482989	FXPR01000001-FXPR01000466
14936_2#22	406764	54.6	5540031	63	87937	1068379	445932	4	4455394	4026541	ERR775528	HE141	pneumoniae	80.54	GCA_900181465	ERS482991	FXPU01000001-FXPU01000063
14936_2#23	446327	53.9	5715070	91	62802.97	1083537	395440	5	4888474	4450403	ERR775529	HE142	pneumoniae	80.81	GCA_900182505	ERS482993	FXSV01000001-FXSV01000091
14936_2#24	416546	53.8	5709021	99	57666.88	939803	371324	6	4566442	4148737	ERR775530	HE143	pneumoniae	80.79	GCA_900181495	ERS482995	FXPV01000001-FXPV01000099
14936_2#25	611265	52.9	5714068	97	58907.92	1081612	393091	5	6675800	6083704	ERR775531	HE144	pneumoniae	79.86	GCA_900181525	ERS482998	FXPZ01000001-FXPZ01000097
14936_2#28	434476	54.3	5618277	98	57329.36	622041	280842	8	4753744	4338838	ERR775534	HE147	pneumoniae	82.46	GCA_900181545	ERS483004	FXQC01000001-FXQC01000098
14936_2#29	365003	46.5	5907999	113	52283.18	855214	435378	5	4001974	3634839	ERR775535	HE148	quasipneum	47.04	GCA_900181565	ERS483006	FXRG01000001-FXRG01000113
14936_2#3	426807	53.9	5620932	73	76999.07	434791	240318	9	4688068	4261721	ERR775509	HE122	pneumoniae	82.82	GCA_900181085	ERS482945	FXOE01000001-FXOE01000073
14936_2#30	401579	52.2	5782932	81	71394.22	918649	350471	5	4391558	3994299	ERR775536	HE149	pneumoniae	80	GCA_900181575	ERS483008	FXQY01000001-FXQY01000081
14936_2#31	408171	52	5777296	84	68777.33	1150701	344618	4	4466332	4048198	ERR775537	HE150	pneumoniae	80.08	GCA_900181585	ERS483010	FXQB01000001-FXQB01000084
14936_2#32	415843	44.9	5906333	114	51809.94	727042	435359	5	4592862	4133655	ERR775538	HE151	quasipneum	47.35	GCA_900181645	ERS483012	FXQO01000001-FXQO01000114
14936_2#33	397962	51.7	5763763	81	71157.57	938360	353243	5	4349690	3976261	ERR775539	HE152	pneumoniae	78.39	GCA_900181655	ERS483013	FXQL01000001-FXQL01000081
14936_2#34	360047	44.7	5903322	107	55171.23	729008	369172	6	3946432	3577653	ERR775540	HE153	quasipneum	47.67	GCA_900181755	ERS483015	FXQV01000001-FXQV01000107
14936_2#35	416057	54.5	5618184	92	61067.22	622082	286149	8	4557562	4153417	ERR775541	HE154	pneumoniae	82.51	GCA_900181675	ERS483017	FXQI01000001-FXQI01000092
14936_2#36	396051	54.7	5620395	99	56771.67	1050993	280888	6	4343210	3955477	ERR775542	HE155	pneumoniae	82.62	GCA_900181735	ERS483019	FXQX01000001-FXQX01000099
14936_2#37	457475	46.4	5800071	109	53211.66	729358	435440	5	5013082	4553636	ERR775543	HE156	quasipneum	46.92	GCA_900181695	ERS483020	FXQP01000001-FXQP01000109
14936_2#38	405229	54.8	5624250	61	92200.82	621536	222759	9	4436420	4055434	ERR775544	HE157	pneumoniae	84.18	GCA_900181715	ERS483022	FXQM01000001-FXQM01000061
14936_2#39	436688	54.5	5625752	58	96995.72	621414	294764	7	4782684	4369542	ERR775545	HE158	pneumoniae	84.5	GCA_900181765	ERS483024	FXQQ01000001-FXQQ01000058
14936_2#4	433247	54.9	5682414	168	33823.89	502653	348875	7	4753634	4234722	ERR775510	HE123	pneumoniae	84.92	GCA_900181095	ERS482949	FXOR01000001-FXOR01000168
14936_2#40	419648	52.3	5762963	86	67011.2	651760	408515	6	4591040	4194805	ERR775546	HE159	pneumoniae	79.16	GCA_900181835	ERS483026	FXRC01000001-FXRC01000086
14936_2#41	395160	45.6	5796431	102	56827.75	854965	305369	6	4326390	3931044	ERR775547	HE160	quasipneum	46.95	GCA_900181785	ERS483027	FXRE01000001-FXRE01000102
14936_2#42	388810	47.9	5909595	140	42211.39	618633	347574	7	4249448	3861727	ERR775548	HE161	pneumoniae	78.87	GCA_900181805	ERS483029	FXQR01000001-FXQR01000140
14936_2#43	382588	47.1	5800019	105	55238.28	854913	489293	5	4197270	3798635	ERR775549	HE162	quasipneum	46.83	GCA_900181865	ERS483031	FXRI01000001-FXRI01000105
14936_2#44	537176	45.6	5802274	98	59206.88	727235	435367	5	5884128	5352362	ERR775550	HE163	quasipneum	46.82	GCA_900181795	ERS483033	FXRB01000001-FXRB01000098
14936_2#45	440013	46.4	5804470	108	53745.09	855108	435241	5	4827716	4387801	ERR775551	HE164	quasipneum	46.82	GCA_900181845	ERS483034	FXRD01000001-FXRD01000108
14936_2#46	395152	45.6	5801945	111	52269.77	727301	438179	5	4323718	3936969	ERR775552	HE165	quasipneum	47.43	GCA_900181885	ERS483036	FXRL01000001-FXRL01000111
14936_2#47	422090	47.8	5774611	227	25438.81	854763	395009	5	4625818	4194462	ERR775553	HE166	quasipneum	46.11	GCA_900181905	ERS483038	FXRJ01000001-FXRJ01000227

Lane	Total yield (k	Matches to r	Total length	No contigs	Average contig length	Largest contig	N50	Contigs in N50	Total raw reads	Reads mapped	Accession	Strain	species	kraken % ma	Assembly accession	Sample accession	Scaffolds accessions
14936_2#48	412738	47.6	5794293	111	52200.84	855816	394344	5	4527854	4115514	ERR775554	HE167	quasipneum	46.61	GCA_900181935	ERS483040	FXRO01000001-FXRO01000111
14936_2#49	458000	47.7	5802667	111	52276.28	854905	435248	5	5025164	4567692	ERR775555	HE168	quasipneum	46.51	GCA_900181925	ERS483042	FXRN01000001-FXRN01000111
14936_2#5	411995	56.1	5522512	81	68179.16	977145	326247	6	4522468	4111612	ERR775511	HE124	pneumoniae	82.38	GCA_900181235	ERS482952	FXPE01000001-FXPE01000081
14936_2#50	384507	45.9	5803923	107	54242.27	854991	435400	5	4218562	3830008	ERR775556	HE169	quasipneum	47.32	GCA_900181915	ERS483045	FXRM01000001-FXRM01000107
14936_2#51	398066	47.4	5798653	105	55225.27	855020	435272	5	4367940	3959681	ERR775557	HE170	quasipneum	46.51	GCA_900181945	ERS483047	FXRP01000001-FXRP01000105
14936_2#52	385626	48.2	5654968	277	20415.05	727178	307974	6	4232974	3837806	ERR775558	HE171	quasipneum	46.2	GCA_900181955	ERS483049	FXRQ01000001-FXRQ01000277
14936_2#53	370194	47.8	5791731	141	41076.11	728658	310882	6	4063984	3680961	ERR775559	HE172	quasipneum	46.32	GCA_900181965	ERS483051	FXRR01000001-FXRR01000141
14936_2#54	357478	45.4	5793038	120	48275.32	726891	435419	5	3917534	3547867	ERR775560	HE173	quasipneum	47.15	GCA_900181975	ERS483052	FXRS01000001-FXRS01000120
14936_2#55	384148	46.3	5800326	107	54208.65	855052	392049	5	4214642	3826886	ERR775561	HE174	quasipneum	47	GCA_900182515	ERS483054	FXSU01000001-FXSU01000107
14936_2#56	406713	46	5802277	98	59206.91	855021	435415	5	4469978	4044436	ERR775562	HE175	quasipneum	46.43	GCA_900181995	ERS483056	FXRV01000001-FXRV01000098
14936_2#57	436195	47.8	5750625	271	21220.02	727377	435242	5	4798822	4332923	ERR775563	HE176	quasipneum	46.07	GCA_900182015	ERS483058	FXRW01000001-FXRW01000271
14936_2#58	382214	46.7	5806310	112	51842.05	727215	308070	6	4190894	3811643	ERR775564	HE177	quasipneum	46.85	GCA_900182005	ERS483061	FXSC01000001-FXSC01000112
14936_2#6	428365	53.6	5621347	64	87833.55	621549	260444	8	4704276	4277684	ERR775512	HE125	pneumoniae	82.2	GCA_900181165	ERS482954	FXOS01000001-FXOS01000064
14936_2#60	404700	48.4	5559394	77	72199.92	855071	414684	5	4441884	4038275	ERR775566	HE179	quasipneum	45.94	GCA_900182035	ERS483065	FXRY01000001-FXRY01000077
14936_2#61	399550	46.2	5803381	105	55270.3	855020	435397	5	4381414	3979345	ERR775567	HE180	quasipneum	46.71	GCA_900182085	ERS483068	FXSB01000001-FXSB01000105
14936_2#63	367704	48.2	5556700	75	74089.33	854996	575064	4	4032526	3660094	ERR775569	HE182	quasipneum	46.03	GCA_900182125	ERS483072	FXSJ01000001-FXSJ01000075
14936_2#64	386232	48.3	5559254	76	73148.08	855249	422003	5	4237450	3849378	ERR775570	HE183	quasipneum	46.03	GCA_900182135	ERS483075	FXSI01000001-FXSI01000076
14936_2#65	394876	45.8	5796822	111	52223.62	640375	305429	7	4326586	3929665	ERR775571	HE184	quasipneum	47.33	GCA_900182175	ERS483076	FXSN01000001-FXSN01000111
14936_2#67	375411	48.3	5561287	77	72224.51	855199	575037	4	4120454	3745576	ERR775573	HE186	quasipneum	46.05	GCA_900182205	ERS483080	FXSP01000001-FXSP01000077
14936_2#68	402611	46.5	5796249	98	59145.4	855104	443283	5	4421416	4012092	ERR775574	HE187	quasipneum	46.84	GCA_900182185	ERS483081	FXSH01000001-FXSH01000098
14936_2#69	384588	46.6	5800838	106	54724.89	854979	394309	5	4219436	3829485	ERR775575	HE188	quasipneum	46.63	GCA_900182145	ERS483083	FXSM01000001-FXSM01000106
14936_2#70	354984	47.5	5798061	113	51310.27	727030	305464	6	3890602	3532034	ERR775576	HE189	quasipneum	46.41	GCA_900182195	ERS483085	FXSK01000001-FXSK01000113
14936_2#71	392646	45.4	5800430	110	52731.18	854964	435402	5	4298258	3909862	ERR775577	HE190	quasipneum	47.08	GCA_900182235	ERS483087	FXSS01000001-FXSS01000110
14936_2#72	359928	47.8	5558972	83	66975.57	855020	583235	4	3949282	3556299	ERR775578	HE191	quasipneum	46.26	GCA_900180895	ERS482879	FXNR01000001-FXNR01000083
14936_2#73	354639	56.5	5568042	68	81882.97	763294	296819	7	3890464	3528485	ERR775579	HE192	pneumoniae	84.88	GCA_900180955	ERS482882	FXNQ01000001-FXNQ01000068
14936_2#74	332677	45.4	5799941	110	52726.74	855197	305277	5	3656466	3304237	ERR775580	HE193	quasipneum	47.04	GCA_900180915	ERS482885	FXMK01000001-FXMK01000110
14936_2#75	351293	48.4	5560632	85	65419.2	727028	435405	5	3854104	3500372	ERR775581	HE194	quasipneum	46.07	GCA_900180565	ERS482889	FXNF01000001-FXNF01000085
14936_2#76	409456	46.9	5803017	106	54745.44	855041	435422	5	4486932	4076640	ERR775582	HE195	quasipneum	46.53	GCA_900180725	ERS482893	FXMO01000001-FXMO01000106
14936_2#77	380277	46.5	5800804	115	50441.77	727186	267610	6	4169660	3788387	ERR775583	HE196	quasipneum	46.63	GCA_900180805	ERS482897	FXMZ01000001-FXMZ01000115
14936_2#78	318118	48.2	5708864	307	18595.65	855152	443444	5	3496132	3159074	ERR775584	HE197	quasipneum	46.3	GCA_900180855	ERS482901	FXNV01000001-FXNV01000307
14936_2#79	349977	48.1	5560773	73	76174.97	854942	586572	4	3840130	3471691	ERR775585	HE198	quasipneum	46.12	GCA_900180585	ERS482904	FXML01000001-FXML01000073
14936_2#80	369490	58.3	5468924	300	18229.75	763132	342789	6	4055994	3629563	ERR775586	HE199	pneumoniae	84.57	GCA_900180745	ERS482909	FXNT01000001-FXNT01000300
14936_2#81	406549	48.2	5557181	77	72171.18	728256	435359	5	4461006	4050268	ERR775587	HE200	quasipneum	46.04	GCA_900180865	ERS482912	FXMM01000001-FXMM01000077
14936_2#82	354924	44.8	5799408	111	52246.92	727275	267733	7	3983414	3543559	ERR775588	HE201	quasipneum	46.24	GCA_900180635	ERS482916	FXMJ01000001-FXMJ01000111
14936_2#83	384751	46.7	5908651	111	53231.09	727216	309662	6	4216396	3832950	ERR775589	HE202	quasipneum	45.81	GCA_900180875	ERS482919	FXNH01000001-FXNH01000111
14936_2#84	390536	50.3	5944027	123	48325.42	1040084	219817	7	4271246	3898561	ERR775590	HE203	pneumoniae	79.28	GCA_900180885	ERS482922	FXNG01000001-FXNG01000123
14936_2#86	377192	46.6	5782952	111	52098.67	727249	305504	6	4151378	3755816	ERR775592	HE205	quasipneum	46.82	GCA_900180755	ERS482928	FXMN01000001-FXMN01000111
14936_2#87	356352	47.8	5748552	93	61812.39	854989	394988	5	3909660	3545287	ERR775593	HE206	quasipneum	46.6	GCA_900180685	ERS482932	FXNU01000001-FXNU01000093
14936_2#88	398787	55.6	5549868	58	95687.38	1068609	451754	4	4372374	3983156	ERR775594	HE207	pneumoniae	81.6	GCA_900180935	ERS482935	FXNA01000001-FXNA01000058
14936_2#89	418445	55.7	5548825	65	85366.54	1068954	451652	4	4590716	4181291	ERR775595	HE208	pneumoniae	81.67	GCA_900181005	ERS482938	FXNZ01000001-FXNZ01000065
14936_2#9	371320	53.8	5619248	73	76976	621503	258378	8	4066812	3709619	ERR775515	HE128	pneumoniae	82.81	GCA_900181255	ERS482962	FXOQ01000001-FXOQ01000073
14936_2#90	382459	47.1	5799482	103	56305.65	855045	435499	5	4203208	3798784	ERR775596	HE209	quasipneum	46.71	GCA_900181055	ERS482941	FXOG01000001-FXOG01000103
14936_2#91	399268	47.4	5801328	104	55782	731043	395703	6	4380718	3979055	ERR775597	HE210	quasipneum	46.42	GCA_900181045	ERS482944	FXOJ01000001-FXOJ01000104
14936_2#92	368969	46.8	5804884	108	53748.93	855198	435397	5	4065566	3679136	ERR775598	HE211	quasipneum	46.87	GCA_900181065	ERS482947	FXOK01000001-FXOK01000108
14936_2#93	403152	46.9	5798326	107	54189.96	855030	435298	5	4420436	4014142	ERR775599	HE212	quasipneum	46.43	GCA_900181145	ERS482950	FXOF01000001-FXOF01000107
14936_2#94	406542	53	5796322	106	54682.28	632468	232169	8	4439764	4050724	ERR775600	HE213	pneumoniae	83.71	GCA_900181125	ERS482953	FXOC01000001-FXOC01000106
14936_2#95	421117	53	5796043	99	58545.89	871832	231390	8	4599644	4200443	ERR775601	HE214	pneumoniae	83.7	GCA_900181135	ERS482956	FXOO01000001-FXOO01000099
14936_3#1	478894	46.6	5577442	85	65616.96	789167	420137	5	5236712	4592158	ERR775602	HE025	quasipneum	45.92	GCA_900181105	ERS482948	FXOI01000001-FXOI01000085
14936_3#10	385816	47	5577033	78	71500.42	1223633	413281	4	4237344	3810331	ERR775611	HE034	quasipneum	44.84	GCA_900181225	ERS482968	FXOX01000001-FXOX01000078
14936_3#11	408719	42.5	5868296	123	47709.72	1919445	1046637	2	4462196	4027444	ERR775612	HE035	quasipneum	49.32	GCA_900181265	ERS482970	FXPC01000001-FXPC01000123
14936_3#13	430793	50.5	5887789	113	52104.33	621465	194120	9	4716508	4277765	ERR775614	HE037	pneumoniae	81.45	GCA_900181375	ERS482974	FXPA01000001-FXPA01000113
14936_3#14	472506	50.4	5879750	123	47802.85	621518	270351	8	5155988	4676498	ERR775615	HE038	pneumoniae	81.46	GCA_900181395	ERS482976	FXPN01000001-FXPN01000123
14936_3#15	433029	51.2	5884558	109	53986.77	621502	227853	9	4739942	4300070	ERR775616	HE039	pneumoniae	81.65	GCA_900181355	ERS482977	FXOY01000001-FXOY01000109
14936_3#17	408003	55.1	5599463	40	139986.58	974167	427488	4	4456354	4053571	ERR775618	HE041	pneumoniae	81.65	GCA_900181425	ERS482981	FXPO01000001-FXPO01000040
14936_3#18	373837	53.2	5577295	72	77462.43	797889	238039	7	4094450	3699834	ERR775619	HE042	pneumoniae	83.39	GCA_900181335	ERS482983	FXPK01000001-FXPK01000072
14936_3#19	421293	53.8	5579381	73	76429.88	1014190	208075	7	4614146	4171314	ERR775620	HE043	pneumoniae	83.8	GCA_900181325	ERS482984	FXOV01000001-FXOV01000073
14936_3#2	429915	55.4	5633959	68	82852.34	1598918	758032	3	4711818	4263371	ERR775603	HE026	pneumoniae	82.22	GCA_900181175	ERS482951	FXOL01000001-FXOL01000068
14936_3#20	406330	55.6	5603402	41	136668.34	885633	441594	4	4450072	4042410	ERR775621	HE044	pneumoniae	81.88	GCA_900181385	ERS482986	FXPP01000001-FXPP01000041
14936_3#21	431413	53.5	5834019	69	84551	916800	227159	8	4728240	4291770	ERR775622	HE045	pneumoniae	82.13	GCA_900181405	ERS482988	FXPM01000001-FXPM01000069
14936_3#22	439226	55.9	5601827	31	180704.1	794065	459232	5	4804856	4368534	ERR775623	HE046	pneumoniae	82.07	GCA_900181435	ERS482990	FXPH01000001-FXPH01000031
14936_3#23	382051	51.7	5828568	86	67774.05	621398	200556	10	4181540	3792105	ERR775624	HE047	pneumoniae	81.09	GCA_900181475	ERS482992	FXPT01000001-FXPT01000086
14936_3#24	389582	56	5602458	34	164778.18	945174	478715	4	4274020	3876159	ERR775625	HE048	pneumoniae	81.94	GCA_900181485	ERS482994	FXPW01000001-FXPW01000034
14936_3#25	404604	52.7	5830942	81	71986.94	763737	263290	8	4419818	4019686	ERR775626	HE049	pneumoniae	81.71	GCA_900181505	ERS482996	FXPX01000001-FXPX01000081
14936_3#26	386882	51.3	5923089	101	58644.45	1061125	514389	4	4226386	3827778	ERR775627	HE050	pneumoniae	81.19	GCA_900181515	ERS482997	FXPY01000001-FXPY01000101
14936_3#27	410733	50.7	5910451	124	47664.93	1061285	404544	5	4481298	4055197	ERR775628	HE051	pneumoniae	80.9	GCA_900181555	ERS482999	FXQA01000001-FXQA01000124
14936_3#28	372651	51.1	5921255	107	55338.83	1062164	479124	4	4072670	3691554	ERR775629	HE052	pneumoniae	81	GCA_900181535	ERS483001	FXQD01000001-FXQD01000107
14936_3#29	389594	57.7	5477690	37	148045.68	1620711	489603	3	4276256	3873847	ERR775630	HE053	pneumoniae	84.73	GCA_900181605	ERS483002	FXQE01000001-FXQE01000037
14936_3#3	457986	55.8	5458676	35	155962.17	1864156	747017	3	5021706	4556600	ERR775604	HE027	pneumoniae	84.49	GCA_900181205	ERS482955	FXOT01000001-FXOT01000035

Lane	Total yield (k	Matches to r	Total length	No contigs	Average contig length	Largest contig	N50	Contigs in N50	Total raw reads	Reads mapped	Accession	Strain	species	kraken % ma	Assembly accession	Sample accession	Scaffolds accessions
14936_3#30	395627	57.1	5475270	35	156436.29	1621549	427678	3	4345142	3932168	ERR775631	HE054	pneumoniae	83.95	GCA_900181665	ERS483005	FXQJ01000001-FXQJ01000035
14936_3#31	358333	56.3	5571190	70	79588.43	489334	265681	8	3929610	3544305	ERR775632	HE055	pneumoniae	83.82	GCA_900181615	ERS483007	FXQF01000001-FXQF01000070
14936_3#32	399715	55.6	5560280	69	80583.77	626825	265473	8	4414508	3965718	ERR775633	HE056	pneumoniae	83.58	GCA_900181595	ERS483009	FXQN01000001-FXQN01000069
14936_3#33	400626	50	5911708	127	46548.88	1293343	405118	4	4363706	3953194	ERR775634	HE057	pneumoniae	80.64	GCA_900181625	ERS483011	FXQH01000001-FXQH01000127
14936_3#34	358132	52.3	5830021	85	68588.48	622103	151828	10	3920118	3528640	ERR775635	HE058	pneumoniae	81.97	GCA_900181635	ERS483014	FXQG01000001-FXQG01000085
14936_3#35	407987	49.9	5856374	141	41534.57	655575	323450	7	4446758	3968291	ERR775636	HE059	pneumoniae	78.01	GCA_900181705	ERS483016	FXQS01000001-FXQS01000141
14936_3#36	353675	53.2	5666442	58	97697.28	768454	351693	6	3865428	3511490	ERR775637	HE060	pneumoniae	79.15	GCA_900181685	ERS483018	FXQK01000001-FXQK01000058
14936_3#37	369031	53.1	5670687	45	126015.27	774894	454809	5	4041580	3669154	ERR775638	HE061	pneumoniae	79.23	GCA_900181745	ERS483021	FXQT01000001-FXQT01000045
14936_3#38	391931	53	5666743	55	103031.69	768228	265185	7	4292384	3874180	ERR775639	HE062	pneumoniae	79.29	GCA_900181725	ERS483023	FXQU01000001-FXQU01000055
14936_3#39	382225	56.3	5561920	59	94269.83	1117075	299164	6	4197898	3745410	ERR775640	HE063	pneumoniae	83.82	GCA_900181815	ERS483025	FXQW01000001-FXQW01000059
14936_3#4	454480	55.3	5633021	69	81637.99	1598373	758113	3	4989220	4519600	ERR775605	HE028	pneumoniae	82.17	GCA_900181195	ERS482958	FXOP01000001-FXOP01000069
14936_3#40	400899	53.1	5665420	52	108950.38	769122	372873	5	4387848	3986599	ERR775641	HE064	pneumoniae	79.29	GCA_900181825	ERS483028	FXQZ01000001-FXQZ01000052
14936_3#41	397698	55.3	5472501	60	91208.35	622176	294452	7	4351224	3951977	ERR775642	HE065	pneumoniae	82.45	GCA_900181855	ERS483030	FXRF01000001-FXRF01000060
14936_3#42	366707	53	5666084	55	103019.71	768499	353851	6	4018684	3642518	ERR775643	HE066	pneumoniae	79.15	GCA_900181775	ERS483032	FXRA01000001-FXRA01000055
14936_3#43	383748	57.4	5426310	48	113048.12	899939	384747	5	4207550	3810335	ERR775644	HE067	pneumoniae	84.16	GCA_900181895	ERS483035	FXRH01000001-FXRH01000048
14936_3#44	350944	57	5426581	52	104357.33	899974	415733	4	3856836	3479536	ERR775645	HE068	pneumoniae	83.7	GCA_900181875	ERS483037	FXRK01000001-FXRK01000052
14936_3#5	452258	46.4	5642975	106	53235.61	1223536	394008	4	4956376	4471037	ERR775606	HE029	quasipneum	44.54	GCA_900181155	ERS482959	FXON01000001-FXON01000106
14936_3#52	339812	56.2	5442296	33	164918.06	874876	342552	6	3730006	3374594	ERR775653	HE076	pneumoniae	81.82	GCA_900181985	ERS483053	FXRT01000001-FXRT01000033
14936_3#56	403205	52.8	5657666	72	78578.69	621374	210961	9	4430608	4004785	ERR775657	HE080	pneumoniae	81.55	GCA_900182045	ERS483060	FXRX01000001-FXRX01000072
14936_3#57	412763	54.8	5800739	173	33530.28	622277	227165	9	4528728	4071841	ERR775658	HE081	pneumoniae	82.5	GCA_900182025	ERS483062	FXRU01000001-FXRU01000173
14936_3#58	394953	51.3	5902393	122	48380.27	916616	263393	7	4320740	3915734	ERR775659	HE082	pneumoniae	81.12	GCA_900182055	ERS483064	FXSA01000001-FXSA01000122
14936_3#59	385656	55.3	5707615	84	67947.8	1634127	759759	3	4223618	3814360	ERR775660	HE083	pneumoniae	81.75	GCA_900182065	ERS483066	FXRZ01000001-FXRZ01000084
14936_3#6	464440	55.7	5635394	72	78269.36	1599946	479013	3	5086682	4613529	ERR775607	HE030	pneumoniae	82.47	GCA_900181215	ERS482961	FXOZ01000001-FXOZ01000072
14936_3#60	354953	53.4	5769607	115	50170.5	602522	199249	10	3880890	3426860	ERR775661	HE084	pneumoniae	81.59	GCA_900182075	ERS483067	FXSD01000001-FXSD01000115
14936_3#62	349632	57.5	5428654	50	108573.08	1065965	391245	4	3873596	3476867	ERR775663	HE086	pneumoniae	82.73	GCA_900182095	ERS483071	FXSF01000001-FXSF01000050
14936_3#63	380446	52.2	5689642	114	49909.14	427440	176713	10	4167368	3732614	ERR775664	HE087	pneumoniae	79.42	GCA_900182105	ERS483073	FXSG01000001-FXSG01000114
14936_3#64	402501	52.1	5881572	108	54459	622157	222956	9	4398804	3997526	ERR775665	HE088	pneumoniae	81.72	GCA_900182115	ERS483074	FXSE01000001-FXSE01000108
14936_3#65	404630	54.8	5580811	58	96220.88	622069	311822	6	4431430	3985089	ERR775666	HE089	pneumoniae	82.57	GCA_900182155	ERS483077	FXSL01000001-FXSL01000058
14936_3#68	380409	58.3	5427667	45	120614.82	797279	448913	5	4178180	3783015	ERR775669	HE092	pneumoniae	83.29	GCA_900182165	ERS483084	FXSO01000001-FXSO01000045
14936_3#7	443219	47	5585791	82	68119.4	1164344	393504	4	4867136	4387444	ERR775608	HE031	quasipneum	44.97	GCA_900181185	ERS482963	FXPB01000001-FXPB01000082
14936_3#70	373243	53.7	5753956	86	66906.47	1420504	309728	5	4087910	3704295	ERR775671	HE094	pneumoniae	81.99	GCA_900182225	ERS483088	FXSR01000001-FXSR01000086
14936_3#71	408117	57.5	5504384	43	128008.93	1318093	506086	3	4476908	4057574	ERR775672	HE095	pneumoniae	83.97	GCA_900182215	ERS483089	FXSQ01000001-FXSQ01000043
14936_3#72	423664	54.4	5615813	71	79095.96	1150356	480546	4	4640116	4210691	ERR775673	HE096	pneumoniae	79.91	GCA_900180775	ERS482877	FXNK01000001-FXNK01000071
14936_3#73	391170	56.8	5491392	100	54913.92	1388735	379715	4	4284412	3846765	ERR775674	HE097	pneumoniae	83.58	GCA_900180815	ERS482880	FXNI01000001-FXNI01000100
14936_3#74	359361	55	5765666	68	84789.21	621448	222965	9	3946586	3565510	ERR775675	HE098	pneumoniae	83.13	GCA_900180535	ERS482883	FXMD01000001-FXMD01000068
14936_3#76	373836	54.9	5667571	53	106935.3	768271	270885	8	4093440	3718543	ERR775677	HE100	pneumoniae	80.99	GCA_900180845	ERS482887	FXMP01000001-FXMP01000053
14936_3#79	387022	52.6	5800201	76	76318.43	627945	263267	8	4241490	3846741	ERR775680	HE103	pneumoniae	82.35	GCA_900180615	ERS482894	FXNJ01000001-FXNJ01000076
14936_3#8	442252	56.1	5459753	37	147560.89	1392979	748568	3	4846608	4398298	ERR775609	HE032	pneumoniae	84.62	GCA_900181245	ERS482965	FXOU01000001-FXOU01000037
14936_3#80	403024	52.6	5794559	73	79377.52	628130	227018	9	4404242	4006072	ERR775681	HE104	pneumoniae	82.48	GCA_900180785	ERS482896	FXMY01000001-FXMY01000073
14936_3#81	389977	50.2	5789613	84	68923.96	621533	222966	9	4248462	3864631	ERR775682	HE105	pneumoniae	81.68	GCA_900180715	ERS482898	FXNO01000001-FXNO01000084
14936_3#84	371125	56.3	5506640	83	66345.06	1604375	537605	3	4069484	3685508	ERR775685	HE108	pneumoniae	82.9	GCA_900180505	ERS482905	FXNS01000001-FXNS01000083
14936_3#85	370534	53.1	5793293	87	66589.57	621397	194247	10	4060382	3681989	ERR775686	HE109	pneumoniae	82.54	GCA_900180555	ERS482907	FXNN01000001-FXNN01000087
14936_3#86	350706	52.5	5795720	78	74304.1	711119	226881	9	3857326	3482244	ERR775687	HE110	pneumoniae	82.93	GCA_900180705	ERS482910	FXMR01000001-FXMR01000078
14936_3#87	364869	51.7	5796261	78	74311.04	1372151	223435	7	3987688	3625179	ERR775688	HE111	pneumoniae	82.45	GCA_900180575	ERS482913	FXMX01000001-FXMX01000078
14936_3#88	388654	50.8	5873807	130	45183.13	589870	214479	8	4246976	3840027	ERR775689	HE112	pneumoniae	78.68	GCA_900180625	ERS482915	FXME01000001-FXME01000130
14936_3#89	378846	51.2	5800339	77	75329.08	621304	280870	8	4130966	3759722	ERR775690	HE113	pneumoniae	81.98	GCA_900180925	ERS482918	FXMQ01000001-FXMQ01000077
14936_3#9	472707	43.4	5876042	111	52937.32	1918820	1045381	2	5167124	4679289	ERR775610	HE033	quasipneum	48.49	GCA_900181285	ERS482966	FXPJ01000001-FXPJ01000111
14936_3#90	382602	51.9	5795165	80	72439.56	1054042	227431	8	4186218	3798234	ERR775691	HE114	pneumoniae	82.21	GCA_900180765	ERS482921	FXMB01000001-FXMB01000080
14936_3#91	385591	52.2	5794221	78	74284.88	763962	294605	7	4221226	3832146	ERR775692	HE115	pneumoniae	82.33	GCA_900180905	ERS482924	FXMS01000001-FXMS01000078
14936_3#92	400263	52	5798920	88	65896.82	622217	201247	10	4377180	3974319	ERR775693	HE116	pneumoniae	81.91	GCA_900180645	ERS482927	FXMH01000001-FXMH01000088
14936_3#93	413030	53.4	5666451	56	101186.62	768241	351128	6	4513544	4103688	ERR775694	HE117	pneumoniae	79.52	GCA_900180605	ERS482930	FXNP01000001-FXNP01000056
14936_3#94	374885	57.9	5330767	54	98717.91	766072	323297	6	4107948	3723276	ERR775695	HE118	pneumoniae	84.57	GCA_900180515	ERS482933	FXMT01000001-FXMT01000054
14936_3#95	378729	58.1	5329295	49	108761.12	788159	464824	5	4154570	3764680	ERR775696	HE119	pneumoniae	84.74	GCA_900180985	ERS482936	FXNX01000001-FXNX01000049
15277_3#61	1062300	59.3	5481687	83	66044.42	1829216	967146	2	11180518	10873074	ERR846973	HE011	pneumoniae	86.36	GCA_900180825	ERS482908	FXNM01000001-FXNM01000083

Technical Appendix Table 2. GenBank accession numbers of published *Klebsiella pneumoniae* strains included in this analysis

ID	Reference	Sample accession no.	Species	Strain	Accession no.
10315_6#1	Chung The et al. (*1*)	ERS249010	*pneumoniae*	16	ERR349747
10315_6#10	Chung The et al. (*1*)	ERS249019	*pneumoniae*	94	ERR349756
10315_6#11	Chung The et al. (*1*)	ERS249020	*pneumoniae*	98	ERR349757
10315_6#13	Chung The et al. (*1*)	ERS249022	*pneumoniae*	108	ERR349759
10315_6#17	Chung The et al. (*1*)	ERS249026	*pneumoniae*	120	ERR349763
10315_6#18	Chung The et al. (*1*)	ERS249027	*pneumoniae*	126	ERR349764
10315_6#19	Chung The et al. (*1*)	ERS249028	*pneumoniae*	127	ERR349765
10315_6#2	Chung The et al. (*1*)	ERS249011	*pneumoniae*	24	ERR349748
10315_6#20	Chung The et al. (*1*)	ERS249029	*pneumoniae*	131	ERR349766
10315_6#21	Chung The et al. (*1*)	ERS249030	*pneumoniae*	132	ERR349767
10315_6#22	Chung The et al. (*1*)	ERS249031	*pneumoniae*	136	ERR349768
10315_6#23	Chung The et al. (*1*)	ERS249032	*pneumoniae*	137	ERR349769
10315_6#24	Chung The et al. (*1*)	ERS249033	*pneumoniae*	139	ERR349770
10315_6#27	Chung The et al. (*1*)	ERS249036	*pneumoniae*	153	ERR349773
10315_6#28	Chung The et al. (*1*)	ERS249037	*pneumoniae*	157	ERR349774
10315_6#29	Chung The et al. (*1*)	ERS249038	*pneumoniae*	159	ERR349775
10315_6#3	Chung The et al. (*1*)	ERS249012	*pneumoniae*	50	ERR349749
10315_6#31	Chung The et al. (*1*)	ERS249040	*pneumoniae*	170	ERR349777
10315_6#34	Chung The et al. (*1*)	ERS249043	*pneumoniae*	186	ERR349780
10315_6#38	Chung The et al. (*1*)	ERS249047	*pneumoniae*	209	ERR349784
10315_6#39	Chung The et al. (*1*)	ERS249048	*pneumoniae*	212	ERR349785
10315_6#40	Chung The et al. (*1*)	ERS249049	*pneumoniae*	214	ERR349786
10315_6#41	Chung The et al. (*1*)	ERS249050	*pneumoniae*	215	ERR349787
10315_6#42	Chung The et al. (*1*)	ERS249051	*pneumoniae*	225	ERR349788
10315_6#43	Chung The et al. (*1*)	ERS249052	*pneumoniae*	230	ERR349789
10315_6#44	Chung The et al. (*1*)	ERS249053	*pneumoniae*	234	ERR349790
10315_6#45	Chung The et al. (*1*)	ERS249054	*pneumoniae*	237	ERR349791
10315_6#49	Chung The et al. (*1*)	ERS249058	*pneumoniae*	270	ERR349795
10315_6#5	Chung The et al. (*1*)	ERS249014	*pneumoniae*	72	ERR349751
10315_6#51	Chung The et al. (*1*)	ERS249060	*pneumoniae*	273	ERR349797
10315_6#52	Chung The et al. (*1*)	ERS249061	*pneumoniae*	276	ERR349798
10315_6#53	Chung The et al. (*1*)	ERS249062	*pneumoniae*	281	ERR349799
10315_6#54	Chung The et al. (*1*)	ERS249063	*pneumoniae*	283	ERR349800
10315_6#57	Chung The et al. (*1*)	ERS249066	*pneumoniae*	305	ERR349803
10315_6#58	Chung The et al. (*1*)	ERS249067	*pneumoniae*	315	ERR349804
10315_6#59	Chung The et al. (*1*)	ERS249068	*pneumoniae*	320	ERR349805
10315_6#6	Chung The et al. (*1*)	ERS249015	*pneumoniae*	80	ERR349752
10315_6#60	Chung The et al. (*1*)	ERS249069	*pneumoniae*	321	ERR349806
10315_6#61	Chung The et al. (*1*)	ERS249070	*pneumoniae*	329	ERR349807
10315_6#62	Chung The et al. (*1*)	ERS249071	*pneumoniae*	352	ERR349808
10315_6#65	Chung The et al. (*1*)	ERS249074	*pneumoniae*	405	ERR349811
10315_6#66	Chung The et al. (*1*)	ERS249075	*pneumoniae*	412	ERR349812
10315_6#67	Chung The et al. (*1*)	ERS249076	*pneumoniae*	420	ERR349813
10315_6#68	Chung The et al. (*1*)	ERS249077	*pneumoniae*	422	ERR349814
10315_6#69	Chung The et al. (*1*)	ERS249078	*pneumoniae*	424	ERR349815
10315_6#7	Chung The et al. (*1*)	ERS249016	*pneumoniae*	81	ERR349753
10315_6#70	Chung The et al. (*1*)	ERS249079	*pneumoniae*	426	ERR349816
10315_6#71	Chung The et al. (*1*)	ERS249080	*pneumoniae*	432	ERR349817
10315_6#72	Chung The et al. (*1*)	ERS249081	*pneumoniae*	434	ERR349818
10315_6#73	Chung The et al. (*1*)	ERS249082	*pneumoniae*	435	ERR349819
10315_6#74	Chung The et al. (*1*)	ERS249083	*pneumoniae*	441	ERR349820
10315_6#75	Chung The et al. (*1*)	ERS249084	*pneumoniae*	446	ERR349821
10315_6#76	Chung The et al. (*1*)	ERS249085	*pneumoniae*	448	ERR349822
10315_6#77	Chung The et al. (*1*)	ERS249086	*pneumoniae*	458	ERR349823
10315_6#78	Chung The et al. (*1*)	ERS249087	*pneumoniae*	482	ERR349824
10315_6#79	Chung The et al. (*1*)	ERS249088	*pneumoniae*	484	ERR349825
10315_6#8	Chung The et al. (*1*)	ERS249017	*pneumoniae*	86	ERR349754
10315_6#80	Chung The et al. (*1*)	ERS249089	*pneumoniae*	486	ERR349826
10315_6#82	Chung The et al. (*1*)	ERS249091	*pneumoniae*	501	ERR349828
10315_6#87	Chung The et al. (*1*)	ERS249096	*pneumoniae*	519	ERR349833
10315_6#88	Chung The et al. (*1*)	ERS249097	*pneumoniae*	524	ERR349834
10315_6#89	Chung The et al. (*1*)	ERS249098	*pneumoniae*	526	ERR349835
10315_6#9	Chung The et al. (*1*)	ERS249018	*pneumoniae*	93	ERR349755
10315_6#92	Chung The et al. (*1*)	ERS249101	*pneumoniae*	539	ERR349838
10315_6#93	Chung The et al. (*1*)	ERS249102	*pneumoniae*	540	ERR349839
10315_6#95	Chung The et al. (*1*)	ERS249104	*pneumoniae*	544	ERR349841

ID	Reference	Sample accession no.	Species	Strain	Accession no.
10315_6#96	Chung The et al. (*1*)	ERS249105	*pneumoniae*	550	ERR349842
10356_5#76	Chung The et al. (*1*)	ERS249106	*pneumoniae*	558	ERR349843
10356_5#77	Chung The et al. (*1*)	ERS249107	*pneumoniae*	567	ERR349844
10356_5#78	Chung The et al. (*1*)	ERS249108	*pneumoniae*	568	ERR349845
10356_5#79	Chung The et al. (*1*)	ERS249109	*pneumoniae*	570	ERR349846
10356_5#80	Chung The et al. (*1*)	ERS249110	*pneumoniae*	573	ERR349847
10356_5#81	Chung The et al. (*1*)	ERS249111	*pneumoniae*	574	ERR349848
10356_5#82	Chung The et al. (*1*)	ERS249112	*pneumoniae*	577	ERR349849
10356_5#85	Chung The et al. (*1*)	ERS249115	*pneumoniae*	586	ERR349852
10356_5#86	Chung The et al. (*1*)	ERS249116	*pneumoniae*	588	ERR349853
10356_5#87	Chung The et al. (*1*)	ERS249117	*pneumoniae*	599	ERR349854
9878_1#11	Chung The et al. (*1*)	ERS237577	*pneumoniae*	587	ERR317538
9878_1#12	Chung The et al. (*1*)	ERS237578	*pneumoniae*	610	ERR317539
9878_1#2	Chung The et al. (*1*)	ERS237568	*pneumoniae*	79	ERR317529
9878_1#3	Chung The et al. (*1*)	ERS237569	*pneumoniae*	85	ERR317530
9878_1#4	Chung The et al. (*1*)	ERS237570	*pneumoniae*	128	ERR317531
9878_1#5	Chung The et al. (*1*)	ERS237571	*pneumoniae*	133	ERR317532
9878_1#6	Chung The et al. (*1*)	ERS237572	*pneumoniae*	146	ERR317533
9878_1#8	Chung The et al. (*1*)	ERS237574	*pneumoniae*	442	ERR317535
9878_1#9	Chung The et al. (*1*)	ERS237575	*pneumoniae*	478	ERR317536
5193_7#7	Holt et al. (*2*)	ERS011884	*pneumoniae*	QMP B2-252	ERR025536
5151_6#8	Holt et al. (*2*)	ERS011957	*pneumoniae*	QMP M1-378	ERR025160
5235_2#6	Holt et al. (*2*)	ERS011991	*pneumoniae*	QMP M1-029	ERR025613
5197_8#7	Holt et al. (*2*)	ERS011896	*pneumoniae*	QMP B2-282	ERR025588
5151_2#11	Holt et al. (*2*)	ERS011924	*pneumoniae*	QMP B2-344	ERR025113
5151_3#5	Holt et al. (*2*)	ERS011930	*pneumoniae*	QMP M1-030	ERR025131
5235_3#4	Holt et al. (*2*)	ERS012001	*pneumoniae*	QMP M2-654	ERR025624
5235_3#1	Holt et al. (*2*)	ERS011998	*pneumoniae*	QMP M2-484	ERR025618
5151_3#6	Holt et al. (*2*)	ERS011931	*pneumoniae*	QMP M1-031	ERR025132
5235_2#8	Holt et al. (*2*)	ERS011993	*pneumoniae*	QMP M2-389	ERR025615
5151_5#11	Holt et al. (*2*)	ERS011948	*pneumoniae*	QMP M1-766	ERR025139
5235_3#7	Holt et al. (*2*)	ERS012004	*pneumoniae*	QMP M2-684	ERR025627
5235_3#2	Holt et al. (*2*)	ERS011999	*pneumoniae*	QMP M2-488	ERR025622
5151_6#7	Holt et al. (*2*)	ERS011956	*pneumoniae*	QMP M1-376	ERR025159
5151_6#6	Holt et al. (*2*)	ERS011955	*pneumoniae*	QMP M1-375	ERR025158
5151_3#10	Holt et al. (*2*)	ERS011935	*pneumoniae*	QMP M1-051	ERR025125
5197_7#7	Holt et al. (*2*)	ERS011872	*pneumoniae*	QMP B2-248	ERR025575
5151_6#1	Holt et al. (*2*)	ERS011950	*pneumoniae*	QMP M1-200	ERR025150
5151_5#7	Holt et al. (*2*)	ERS011944	*pneumoniae*	QMP M1-728	ERR025146
5235_3#11	Holt et al. (*2*)	ERS012008	*pneumoniae*	QMP Z4-702	ERR025620
5151_5#8	Holt et al. (*2*)	ERS011945	*pneumoniae*	QMP M1-761	ERR025147
5299_1#2	Holt et al. (*2*)	ERS011963	*pneumoniae*	QMP M1-821	ERR025983
5151_5#2	Holt et al. (*2*)	ERS011939	*pneumoniae*	QMP M1-559	ERR025141
5151_6#5	Holt et al. (*2*)	ERS011954	*pneumoniae*	QMP M1-222	ERR025157
5235_3#12	Holt et al. (*2*)	ERS012009	*pneumoniae*	QMP Z4-724	ERR025621
5151_3#2	Holt et al. (*2*)	ERS011927	*pneumoniae*	QMP B2-563	ERR025128
5151_6#3	Holt et al. (*2*)	ERS011952	*pneumoniae*	QMP M1-218	ERR025155
5151_6#2	Holt et al. (*2*)	ERS011951	*pneumoniae*	QMP M1-217	ERR025154
5151_5#3	Holt et al. (*2*)	ERS011940	*pneumoniae*	QMP M1-560	ERR025142
5193_2#7	Holt et al. (*2*)	ERS011812	*pneumoniae*	QMP B2-176	ERR025484
5299_7#7	Holt et al. (*2*)	ERS012052	*pneumoniae*	QMP Z4-716	ERR025999
5151_6#4	Holt et al. (*2*)	ERS011953	*pneumoniae*	QMP M1-220	ERR025156
5151_3#11	Holt et al. (*2*)	ERS011936	*pneumoniae*	QMP M1-198	ERR025126
5235_6#7	Holt et al. (*2*)	ERS012028	*pneumoniae*	QMP Z4-709	ERR025653
5235_7#7	Holt et al. (*2*)	ERS012040	*pneumoniae*	QMP Z4-712	ERR025666
5193_6#7	Holt et al. (*2*)	ERS011860	*pneumoniae*	QMP B2-228	ERR025523
5299_1#1	Holt et al. (*2*)	ERS011962	*pneumoniae*	QMP M1-781	ERR025979
5193_5#7	Holt et al. (*2*)	ERS011848	*pneumoniae*	QMP B2-223	ERR025510
5151_5#4	Holt et al. (*2*)	ERS011941	*pneumoniae*	QMP M1-561	ERR025143
5151_5#5	Holt et al. (*2*)	ERS011942	*pneumoniae*	QMP M1-562	ERR025144
5299_1#4	Holt et al. (*2*)	ERS011965	*pneumoniae*	QMP M1-826	ERR025985
5299_1#3	Holt et al. (*2*)	ERS011964	*pneumoniae*	QMP M1-822	ERR025984
5151_5#1	Holt et al. (*2*)	ERS011938	*pneumoniae*	QMP M1-557	ERR025137
5197_2#7	Holt et al. (*2*)	ERS011825	*pneumoniae*	QMP B2-211	ERR025562
5235_8#7	Holt et al. (*2*)	ERS011789	*pneumoniae*	QMP B2-090	ERR025678
5193_1#7	Holt et al. (*2*)	ERS011800	*pneumoniae*	QMP B2-170	ERR025471
5193_3#12	Holt et al. (*2*)	ERS011841	*pneumoniae*	D-022-I-b-1	ERR025491
5193_6#2	Holt et al. (*2*)	ERS011855	*pneumoniae*	EW-67-R-MAC	ERR025518

ID	Reference	Sample accession no.	Species	Strain	Accession no.
5193_5#8	Holt et al. (*2*)	ERS011849	*pneumoniae*	EW-20-R-MAG-1	ERR025511
5193_2#8	Holt et al. (*2*)	ERS011813	*pneumoniae*	K21Sp	ERR025485
5193_3#7	Holt et al. (*2*)	ERS011836	*pneumoniae*	K296N	ERR025497
5197_2#10	Holt et al. (*2*)	ERS011827	*pneumoniae*	K261An	ERR025554
5193_3#10	Holt et al. (*2*)	ERS011839	*pneumoniae*	B-013-I-a-2	ERR025489
5193_5#2	Holt et al. (*2*)	ERS011843	*pneumoniae*	015-CN-2	ERR025505
5193_5#9	Holt et al. (*2*)	ERS011850	*pneumoniae*	EW-20-R-MAC-1	ERR025512
5197_2#6	Holt et al. (*2*)	ERS011823	*pneumoniae*	K228An	ERR025561
5193_3#9	Holt et al. (*2*)	ERS011838	*pneumoniae*	A-003-I-a-1	ERR025499
5193_3#4	Holt et al. (*2*)	ERS011833	*pneumoniae*	K280N	ERR025494
5197_2#8	Holt et al. (*2*)	ERS011824	*pneumoniae*	K231An	ERR025563
5193_5#11	Holt et al. (*2*)	ERS011852	*pneumoniae*	EW-33-R-MAC-2	ERR025503
5193_2#12	Holt et al. (*2*)	ERS011817	*pneumoniae*	K77An	ERR025478
5197_2#9	Holt et al. (*2*)	ERS011826	*pneumoniae*	K242An	ERR025564
5193_5#10	Holt et al. (*2*)	ERS011851	*pneumoniae*	EW-29-R-MAG	ERR025502
5197_2#3	Holt et al. (*2*)	ERS011820	*pneumoniae*	K113N	ERR025558
5193_6#3	Holt et al. (*2*)	ERS011856	*pneumoniae*	EW-68-R-MAC-1	ERR025519
5197_2#1	Holt et al. (*2*)	ERS011818	*pneumoniae*	K86N	ERR025553
5193_3#8	Holt et al. (*2*)	ERS011837	*pneumoniae*	K307An	ERR025498
5193_3#5	Holt et al. (*2*)	ERS011834	*pneumoniae*	K282Ax	ERR025495
5193_2#9	Holt et al. (*2*)	ERS011814	*pneumoniae*	K35N	ERR025486
5193_2#11	Holt et al. (*2*)	ERS011816	*pneumoniae*	K53N	ERR025477
5193_3#6	Holt et al. (*2*)	ERS011835	*pneumoniae*	K290N	ERR025496
5193_5#1	Holt et al. (*2*)	ERS011842	*pneumoniae*	D-026-I-b-1	ERR025501
5197_2#11	Holt et al. (*2*)	ERS011828	*pneumoniae*	K262N	ERR025555
5193_6#4	Holt et al. (*2*)	ERS011857	*pneumoniae*	EW-85-R-MAN	ERR025520
5193_5#12	Holt et al. (*2*)	ERS011853	*pneumoniae*	EW-44-R-MAG-1	ERR025504
5197_2#2	Holt et al. (*2*)	ERS011819	*pneumoniae*	K102An	ERR025557
5235_2#3	Holt et al. (*2*)	ERS011988	*pneumoniae*	QMP M1-975	ERR025610
5235_2#2	Holt et al. (*2*)	ERS011987	*pneumoniae*	QMP M1-974	ERR025609
5235_1#11	Holt et al. (*2*)	ERS011984	*pneumoniae*	QMP M1-968	ERR025594
5193_8#8	Holt et al. (*2*)	ERS011909	*pneumoniae*	DU33062/05	ERR025550
5193_8#5	Holt et al. (*2*)	ERS011906	*pneumoniae*	DR5092/05	ERR025547
5193_2#6	Holt et al. (*2*)	ERS011811	*pneumoniae*	UV1714	ERR025483
5193_2#5	Holt et al. (*2*)	ERS011810	*pneumoniae*	UV1625	ERR025482
5193_2#1	Holt et al. (*2*)	ERS011806	*pneumoniae*	UV1172	ERR025475
5193_1#12	Holt et al. (*2*)	ERS011805	*pneumoniae*	UV937	ERR025465
5193_2#2	Holt et al. (*2*)	ERS011807	*pneumoniae*	NCSR101	ERR025479
5235_1#3	Holt et al. (*2*)	ERS011976	*pneumoniae*	QMP M1-892	ERR025597
5299_1#9	Holt et al. (*2*)	ERS011970	*pneumoniae*	QMP M1-885	ERR025990
5299_1#10	Holt et al. (*2*)	ERS011971	*pneumoniae*	QMP M1-886	ERR025980
5299_1#7	Holt et al. (*2*)	ERS011968	*pneumoniae*	QMP M1-882	ERR025988
5299_1#11	Holt et al. (*2*)	ERS011972	*pneumoniae*	QMP M1-887	ERR025981
5235_3#6	Holt et al. (*2*)	ERS012003	*pneumoniae*	QMP M1-860	ERR025626
5235_1#6	Holt et al. (*2*)	ERS011979	*pneumoniae*	QMP M1-896	ERR025600
5299_1#8	Holt et al. (*2*)	ERS011969	*pneumoniae*	QMP M1-884	ERR025989
5235_1#2	Holt et al. (*2*)	ERS011975	*pneumoniae*	QMP M1-891	ERR025596
5151_2#6	Holt et al. (*2*)	ERS011919	*pneumoniae*	DU8882/04	ERR025119
5151_2#3	Holt et al. (*2*)	ERS011916	*pneumoniae*	DM17337/04	ERR025116
5151_2#10	Holt et al. (*2*)	ERS011923	*pneumoniae*	DU46543/08	ERR025112
5193_8#3	Holt et al. (*2*)	ERS011904	*pneumoniae*	DU4033/04	ERR025545
5193_8#6	Holt et al. (*2*)	ERS011907	*pneumoniae*	DU10252/04	ERR025548
5193_8#1	Holt et al. (*2*)	ERS011902	*pneumoniae*	DM23092/04	ERR025540
5193_8#10	Holt et al. (*2*)	ERS011911	*pneumoniae*	DU38032/05	ERR025541
5235_5#6	Holt et al. (*2*)	ERS012015	*pneumoniae*	09-309B	ERR025639
5235_6#2	Holt et al. (*2*)	ERS012023	*pneumoniae*	09-341B	ERR025648
5235_8#8	Holt et al. (*2*)	ERS011790	*pneumoniae*	71B	ERR025679
5235_8#2	Holt et al. (*2*)	ERS011784	*pneumoniae*	08-049B	ERR025673
5193_1#9	Holt et al. (*2*)	ERS011802	*pneumoniae*	08-058D	ERR025473
5235_5#11	Holt et al. (*2*)	ERS012020	*pneumoniae*	09-332B	ERR025633
5150_1#3	Holt et al. (*2*)	ERS005743	*pneumoniae*	AJ049	ERR024822
5150_2#5	Holt et al. (*2*)	ERS005757	*pneumoniae*	AJ056	ERR024837
5150_3#6	Holt et al. (*2*)	ERS005770	*pneumoniae*	AJ158	ERR024851

ID	Reference	Sample accession no.	Species	Strain	Accession no.
5150_5#7	Holt et al. (*2*)	ERS005783	*pneumoniae*	AJ229	ERR025107
5150_2#3	Holt et al. (*2*)	ERS005756	*pneumoniae*	AJ146	ERR024835
5150_2#7	Holt et al. (*2*)	ERS005759	*pneumoniae*	AJ082	ERR024839
5150_3#9	Holt et al. (*2*)	ERS005773	*pneumoniae*	AJ188	ERR024854
5150_5#9	Holt et al. (*2*)	ERS005785	*pneumoniae*	AJ278	ERR025109
5150_1#11	Holt et al. (*2*)	ERS005751	*pneumoniae*	AJ034	ERR024819
5150_1#2	Holt et al. (*2*)	ERS005742	*pneumoniae*	AJ048	ERR024821
5150_5#5	Holt et al. (*2*)	ERS005781	*pneumoniae*	AJ218	ERR025105
5150_3#1	Holt et al (*2*)	ERS005765	*pneumoniae*	AJ155	ERR024843
5150_2#8	Holt et al. (*2*)	ERS005760	*pneumoniae*	AJ083	ERR024840
5150_2#4	Holt et al. (*2*)	ERS005755	*pneumoniae*	AJ148	ERR024836
5150_1#4	Holt et al. (*2*)	ERS005744	*pneumoniae*	AJ054	ERR024823
5150_2#11	Holt et al. (*2*)	ERS005763	*pneumoniae*	AJ097	ERR024832
5150_2#10	Holt et al. (*2*)	ERS005762	*pneumoniae*	AJ094	ERR024831
5150_3#11	Holt et al. (*2*)	ERS005775	*pneumoniae*	AJ205	ERR024845
5150_3#3	Holt et al. (*2*)	ERS005767	*pneumoniae*	AJ211	ERR024848
5193_7#3	Holt et al. (*2*)	ERS011880	*pneumoniae*	U 16821	ERR025532
5193_7#2	Holt et al. (*2*)	ERS011879	*pneumoniae*	U 13792/2	ERR025531
5193_7#1	Holt et al. (*2*)	ERS011878	*pneumoniae*	U 12567	ERR025527
5197_7#8	Holt et al. (*2*)	ERS011873	*pneumoniae*	Pus 15987	ERR025576
5151_5#12	Holt et al. (*2*)	ERS011949	*pneumoniae*	QMP M1-771	ERR025140
5235_3#8	Holt et al. (*2*)	ERS012005	*pneumoniae*	QMP M1-862	ERR025628
5235_3#9	Holt et al. (*2*)	ERS012006	*pneumoniae*	QMP M1-868	ERR025629
5235_3#5	Holt et al. (*2*)	ERS012002	*pneumoniae*	QMP M1-776	ERR025625
5193_8#12	Holt et al. (*2*)	ERS011913	*pneumoniae*	DR19891/02	ERR025543
5193_8#2	Holt et al. (*2*)	ERS011903	*pneumoniae*	DB44834/96	ERR025544
5193_8#4	Holt et al. (*2*)	ERS011905	*pneumoniae*	DB11802/05	ERR025546
5151_2#8	Holt et al. (*2*)	ERS011921	*pneumoniae*	DX259/08	ERR025121
5151_2#5	Holt et al. (*2*)	ERS011918	*pneumoniae*	DB270/04	ERR025118
5151_2#1	Holt et al. (*2*)	ERS011914	*pneumoniae*	DM16912/02	ERR025111
5151_2#2	Holt et al. (*2*)	ERS011915	*pneumoniae*	DM17138/03	ERR025115
5151_2#4	Holt et al. (*2*)	ERS011917	*pneumoniae*	DM11825/05	ERR025117
5193_8#11	Holt et al. (*2*)	ERS011912	*pneumoniae*	DM1159/01	ERR025542
5299_7#1	Holt et al. (*2*)	ERS012046	*pneumoniae*	1824	ERR025992
5299_7#9	Holt et al. (*2*)	ERS012054	*pneumoniae*	1522	ERR026001
5299_7#6	Holt et al. (*2*)	ERS012051	*pneumoniae*	2024	ERR025998
5235_6#11	Holt et al. (*2*)	ERS012032	*pneumoniae*	1517	ERR025646
5299_7#3	Holt et al. (*2*)	ERS012048	*pneumoniae*	1896	ERR025995
5299_7#5	Holt et al. (*2*)	ERS012050	*pneumoniae*	1993	ERR025997
5235_6#12	Holt et al. (*2*)	ERS012033	*pneumoniae*	1523	ERR025647
5235_7#4	Holt et al. (*2*)	ERS012037	*pneumoniae*	1576	ERR025663
5235_7#5	Holt et al. (*2*)	ERS012038	*pneumoniae*	1585	ERR025664
5235_7#6	Holt et al. (*2*)	ERS012039	*pneumoniae*	1586	ERR025665
5299_7#2	Holt et al. (*2*)	ERS012047	*pneumoniae*	1884	ERR025994
5299_7#4	Holt et al. (*2*)	ERS012049	*pneumoniae*	1897	ERR025996
5235_7#8	Holt et al. (*2*)	ERS012041	*pneumoniae*	1612	ERR025667
5235_7#12	Holt et al. (*2*)	ERS012045	*pneumoniae*	1789	ERR025660
5235_7#11	Holt et al. (*2*)	ERS012044	*pneumoniae*	1765	ERR025659
5235_7#9	Holt et al. (*2*)	ERS012042	*pneumoniae*	1753	ERR025668
5235_7#10	Holt et al. (*2*)	ERS012043	*pneumoniae*	1764	ERR025658
5235_8#3	Holt et al. (*2*)	ERS011785	*pneumoniae*	71M	ERR025674
5235_8#6	Holt et al. (*2*)	ERS011788	*pneumoniae*	08-0116m	ERR025677
5235_6#10	Holt et al. (*2*)	ERS012031	*pneumoniae*	805m	ERR025645
5235_6#9	Holt et al. (*2*)	ERS012030	*pneumoniae*	558m	ERR025655
5235_5#5	Holt et al. (*2*)	ERS012014	*pneumoniae*	09-2006m	ERR025638
5235_5#8	Holt et al. (*2*)	ERS012017	*pneumoniae*	09-2072m	ERR025641
5235_5#2	Holt et al. (*2*)	ERS012011	*pneumoniae*	09-2503m	ERR025635
5235_8#4	Holt et al. (*2*)	ERS011786	*pneumoniae*	08-0345m	ERR025675
5235_8#5	Holt et al. (*2*)	ERS011787	*pneumoniae*	24m	ERR025676
5235_6#3	Holt et al. (*2*)	ERS012024	*pneumoniae*	09-2985m	ERR025649
5193_1#4	Holt et al. (*2*)	ERS011797	*pneumoniae*	07-0003m	ERR025468
5235_6#8	Holt et al. (*2*)	ERS012029	*pneumoniae*	2358m	ERR025654
5193_1#8	Holt et al. (*2*)	ERS011801	*pneumoniae*	08-475T	ERR025472
5193_1#6	Holt et al. (*2*)	ERS011799	*pneumoniae*	07-2034m	ERR025470
5193_1#11	Holt et al. (*2*)	ERS011804	*pneumoniae*	08-1177m	ERR025464
5193_1#5	Holt et al. (*2*)	ERS011798	*pneumoniae*	09-0079m	ERR025469
5193_1#1	Holt et al. (*2*)	ERS011794	*pneumoniae*	59M	ERR025462
5235_6#6	Holt et al. (*2*)	ERS012027	*pneumoniae*	620m	ERR025652

ID	Reference	Sample accession no.	Species	Strain	Accession no.
5150_5#3	Holt et al. (*2*)	ERS005779	*pneumoniae*	AJ299	ERR025103
5150_1#5	Holt et al. (*2*)	ERS005745	*pneumoniae*	AJ006	ERR024824
5150_5#1	Holt et al. (*2*)	ERS005777	*pneumoniae*	AJ214	ERR025098
5150_5#10	Holt et al. (*2*)	ERS005786	*pneumoniae*	AJ281	ERR025099
5150_5#11	Holt et al. (*2*)	ERS005787	*pneumoniae*	AJ289	ERR025100
5197_8#11	Holt et al. (*2*)	ERS011900	*pneumoniae*	UI 14245	ERR025581
5197_8#1	Holt et al. (*2*)	ERS011890	*pneumoniae*	UI 6167	ERR025579
5197_7#2	Holt et al. (*2*)	ERS011867	*pneumoniae*	Pus 4878	ERR025570
5193_7#5	Holt et al. (*2*)	ERS011882	*pneumoniae*	UI 522	ERR025534
5193_7#9	Holt et al. (*2*)	ERS011886	*pneumoniae*	UI 3324	ERR025538
5197_8#4	Holt et al. (*2*)	ERS011893	*pneumoniae*	UI 8601	ERR025585
5197_8#12	Holt et al. (*2*)	ERS011901	*pneumoniae*	UI 15398	ERR025582
5197_7#6	Holt et al. (*2*)	ERS011871	*pneumoniae*	Pus 15007	ERR025574
5197_8#2	Holt et al. (*2*)	ERS011891	*pneumoniae*	UI 6717	ERR025583
5197_8#6	Holt et al. (*2*)	ERS011895	*pneumoniae*	UI 10871	ERR025587
5197_7#1	Holt et al. (*2*)	ERS011866	*pneumoniae*	AF 3927	ERR025566
5193_7#6	Holt et al. (*2*)	ERS011883	*pneumoniae*	UI 2213	ERR025535
5197_7#3	Holt et al. (*2*)	ERS011868	*pneumoniae*	Pus 9314/2	ERR025571
5197_8#8	Holt et al. (*2*)	ERS011897	*pneumoniae*	ST 752	ERR025589
5193_6#10	Holt et al. (*2*)	ERS011863	*pneumoniae*	Kp-Miami	ERR025515
5299_7#10	Holt et al. (*2*)	ERS012055	*pneumoniae*	TL125	ERR025993
5151_6#9	Holt et al. (*2*)	ERS011958	*pneumoniae*	QMP M1-406	ERR025161
5235_2#12	Holt et al. (*2*)	ERS011997	*pneumoniae*	QMP M1-418	ERR025608
5235_2#7	Holt et al. (*2*)	ERS011992	*pneumoniae*	QMP M1-414	ERR025614
5235_2#11	Holt et al. (*2*)	ERS011996	*pneumoniae*	QMP M1-407	ERR025607
5151_6#10	Holt et al. (*2*)	ERS011959	*pneumoniae*	QMP M1-413	ERR025151
5151_6#11	Holt et al. (*2*)	ERS011960	*pneumoniae*	QMP M1-415	ERR025152
5235_1#9	Holt et al. (*2*)	ERS011982	*pneumoniae*	QMP M1-965	ERR025603
5235_2#5	Holt et al. (*2*)	ERS011990	*pneumoniae*	QMP M1-980	ERR025612
5235_1#8	Holt et al. (*2*)	ERS011981	*pneumoniae*	QMP M1-964	ERR025602
5235_2#1	Holt et al. (*2*)	ERS011986	*pneumoniae*	QMP M1-972	ERR025605
5235_7#3	Holt et al. (*2*)	ERS012036	*pneumoniae*	1557	ERR025662
5299_7#8	Holt et al. (*2*)	ERS012053	*pneumoniae*	2033	ERR026000
5235_7#2	Holt et al. (*2*)	ERS012035	*pneumoniae*	1555	ERR025661
5235_7#1	Holt et al. (*2*)	ERS012034	*pneumoniae*	1524	ERR025657
5235_8#9	Holt et al. (*2*)	ERS011791	*pneumoniae*	09-286G	ERR025680
5235_6#5	Holt et al. (*2*)	ERS012026	*pneumoniae*	311G	ERR025651
5193_6#9	Holt et al. (*2*)	ERS011862	*pneumoniae*	7085	ERR025525
5193_6#8	Holt et al. (*2*)	ERS011861	*pneumoniae*	206535	ERR025524
5193_6#6	Holt et al. (*2*)	ERS011859	*pneumoniae*	MM50237	ERR025522
5193_6#5	Holt et al. (*2*)	ERS011858	*pneumoniae*	KpV513	ERR025521
5193_6#11	Holt et al. (*2*)	ERS011864	*pneumoniae*	812079	ERR025516
5193_6#12	Holt et al. (*2*)	ERS011865	*pneumoniae*	85997	ERR025517
9263_7#10	Pérez-Vázquez et al. (*3*)	ERS201950	*pneumoniae*	K1039	ERR264510
9263_7#11	Pérez-Vázquez et al. (*3*)	ERS201951	*pneumoniae*	K1057	ERR264511
9263_7#12	Pérez-Vázquez et al. (*3*)	ERS201952	*pneumoniae*	K1202	ERR264512
9263_7#13	Pérez-Vázquez et al. (*3*)	ERS201953	*pneumoniae*	K1232	ERR264513
9263_7#14	Pérez-Vázquez et al. (*3*)	ERS201954	*pneumoniae*	K1299	ERR264514
9263_7#15	Pérez-Vázquez et al. (*3*)	ERS201955	*pneumoniae*	K1119	ERR264515
9263_7#16	Pérez-Vázquez et al. (*3*)	ERS201956	*pneumoniae*	K1288	ERR264516
9263_7#17	Pérez-Vázquez et al. (*3*)	ERS201957	*pneumoniae*	K1344	ERR264517
9263_7#18	Pérez-Vázquez et al. (*3*)	ERS201958	*pneumoniae*	K754	ERR264518
9263_7#19	Pérez-Vázquez et al. (*3*)	ERS201959	*pneumoniae*	K863	ERR264519
9263_7#1	Pérez-Vázquez et al. (*3*)	ERS201941	*pneumoniae*	K750	ERR264501
9263_7#20	Pérez-Vázquez et al. (*3*)	ERS201960	*pneumoniae*	K943	ERR264520
9263_7#21	Pérez-Vázquez et al. (*3*)	ERS201961	*pneumoniae*	K944	ERR264521
9263_7#22	Pérez-Vázquez et al. (*3*)	ERS201962	*pneumoniae*	K954	ERR264522
9263_7#23	Pérez-Vázquez et al. (*3*)	ERS201963	*pneumoniae*	K966	ERR264523
9263_7#24	Pérez-Vázquez et al. (*3*)	ERS201964	*pneumoniae*	K1026	ERR264524
9263_7#25	Pérez-Vázquez et al. (*3*)	ERS201965	*pneumoniae*	K864	ERR264525
9263_7#27	Pérez-Vázquez et al. (*3*)	ERS201967	*pneumoniae*	K922	ERR264527
9263_7#28	Pérez-Vázquez et al. (*3*)	ERS201968	*pneumoniae*	K924	ERR264528
9263_7#29	Pérez-Vázquez et al. (*3*)	ERS201969	*pneumoniae*	K953	ERR264529
9263_7#2	Pérez-Vázquez et al. (*3*)	ERS201942	*pneumoniae*	K791	ERR264502
9263_7#30	Pérez-Vázquez et al. (*3*)	ERS201970	*pneumoniae*	K967	ERR264530
9263_7#31	Pérez-Vázquez et al. (*3*)	ERS201971	*pneumoniae*	K1028	ERR264531
9263_7#32	Pérez-Vázquez et al. (*3*)	ERS201972	*pneumoniae*	K1029	ERR264532
9263_7#33	Pérez-Vázquez et al. (*3*)	ERS201973	*pneumoniae*	K1031	ERR264533

ID	Reference	Sample accession no.	Species	Strain	Accession no.
9263_7#35	Pérez-Vázquez et al. (*3*)	ERS201975	*pneumoniae*	K1047	ERR264535
9263_7#36	Pérez-Vázquez et al. (*3*)	ERS201976	*pneumoniae*	K1049	ERR264536
9263_7#37	Pérez-Vázquez et al. (*3*)	ERS201977	*pneumoniae*	K1050	ERR264537
9263_7#38	Pérez-Vázquez et al. (*3*)	ERS201978	*pneumoniae*	K1061	ERR264538
9263_7#39	Pérez-Vázquez et al. (*3*)	ERS201979	*pneumoniae*	K1063	ERR264539
9263_7#3	Pérez-Vázquez et al. (*3*)	ERS201943	*pneumoniae*	K792	ERR264503
9263_7#40	Pérez-Vázquez et al. (*3*)	ERS201980	*pneumoniae*	K1064	ERR264540
9263_7#41	Pérez-Vázquez et al. (*3*)	ERS201981	*pneumoniae*	K1065	ERR264541
9263_7#42	Pérez-Vázquez et al. (*3*)	ERS201982	*pneumoniae*	K1069	ERR264542
9263_7#43	Pérez-Vázquez et al. (*3*)	ERS201983	*pneumoniae*	K1070	ERR264543
9263_7#44	Pérez-Vázquez et al. (*3*)	ERS201984	*pneumoniae*	K1071	ERR264544
9263_7#45	Pérez-Vázquez et al. (*3*)	ERS201985	*pneumoniae*	K1072	ERR264545
9263_7#46	Pérez-Vázquez et al. (*3*)	ERS201986	*pneumoniae*	K1073	ERR264546
9263_7#47	Pérez-Vázquez et al. (*3*)	ERS201987	*pneumoniae*	K1074	ERR264547
9263_7#48	Pérez-Vázquez et al. (*3*)	ERS201988	*pneumoniae*	K725	ERR264548
9263_7#49	Pérez-Vázquez et al. (*3*)	ERS201989	*pneumoniae*	K727	ERR264549
9263_7#4	Pérez-Vázquez et al. (*3*)	ERS201944	*pneumoniae*	K946	ERR264504
9263_7#50	Pérez-Vázquez et al. (*3*)	ERS201990	*pneumoniae*	K726	ERR264550
9263_7#51	Pérez-Vázquez et al. (*3*)	ERS201991	*pneumoniae*	K730	ERR264551
9263_7#52	Pérez-Vázquez et al. (*3*)	ERS201992	*pneumoniae*	K757	ERR264552
9263_7#53	Pérez-Vázquez et al. (*3*)	ERS201993	*pneumoniae*	K758	ERR264553
9263_7#54	Pérez-Vázquez et al. (*3*)	ERS201994	*pneumoniae*	K760	ERR264554
9263_7#55	Pérez-Vázquez et al. (*3*)	ERS201995	*pneumoniae*	K761	ERR264555
9263_7#56	Pérez-Vázquez et al. (*3*)	ERS201996	*pneumoniae*	K763	ERR264556
9263_7#57	Pérez-Vázquez et al. (*3*)	ERS201997	*pneumoniae*	K796	ERR264557
9263_7#58	Pérez-Vázquez et al. (*3*)	ERS201998	*pneumoniae*	K810	ERR264558
9263_7#59	Pérez-Vázquez et al. (*3*)	ERS201999	*pneumoniae*	K811	ERR264559
9263_7#5	Pérez-Vázquez et al. (*3*)	ERS201945	*pneumoniae*	K804	ERR264505
9263_7#60	Pérez-Vázquez et al. (*3*)	ERS202000	*pneumoniae*	K893	ERR264560
9263_7#61	Pérez-Vázquez et al. (*3*)	ERS202001	*pneumoniae*	K870	ERR264561
9263_7#62	Pérez-Vázquez et al. (*3*)	ERS202002	*pneumoniae*	K871	ERR264562
9263_7#63	Pérez-Vázquez et al. (*3*)	ERS202003	*pneumoniae*	K874	ERR264563
9263_7#64	Pérez-Vázquez et al. (*3*)	ERS202004	*pneumoniae*	K875	ERR264564
9263_7#65	Pérez-Vázquez et al. (*3*)	ERS202005	*pneumoniae*	K879	ERR264565
9263_7#66	Pérez-Vázquez et al. (*3*)	ERS202006	*pneumoniae*	K892	ERR264566
9263_7#67	Pérez-Vázquez et al. (*3*)	ERS202007	*pneumoniae*	K925	ERR264567
9263_7#68	Pérez-Vázquez et al. (*3*)	ERS202008	*pneumoniae*	K935	ERR264568
9263_7#69	Pérez-Vázquez et al. (*3*)	ERS202009	*pneumoniae*	K756	ERR264569
9263_7#6	Pérez-Vázquez et al. (*3*)	ERS201946	*pneumoniae*	K983	ERR264506
9263_7#70	Pérez-Vázquez et al. (*3*)	ERS202010	*pneumoniae*	K809	ERR264570
9263_7#71	Pérez-Vázquez et al. (*3*)	ERS202011	*pneumoniae*	K891	ERR264571
9263_7#72	Pérez-Vázquez et al. (*3*)	ERS202012	*pneumoniae*	K889	ERR264572
9263_7#73	Pérez-Vázquez et al. (*3*)	ERS202013	*pneumoniae*	K876	ERR264573
9263_7#74	Pérez-Vázquez et al. (*3*)	ERS202014	*pneumoniae*	K877	ERR264574
9263_7#75	Pérez-Vázquez et al. (*3*)	ERS202015	*pneumoniae*	K881	ERR264575
9263_7#76	Pérez-Vázquez et al. (*3*)	ERS202016	*pneumoniae*	K882	ERR264576
9263_7#77	Pérez-Vázquez et al. (*3*)	ERS202017	*pneumoniae*	K884	ERR264577
9263_7#7	Pérez-Vázquez et al. (*3*)	ERS201947	*pneumoniae*	K779	ERR264507
9263_7#8	Pérez-Vázquez et al. (*3*)	ERS201948	*pneumoniae*	K956	ERR264508
9263_7#9	Pérez-Vázquez et al. (*3*)	ERS201949	*pneumoniae*	K1035	ERR264509
9517_7#10	Pérez-Vázquez et al. (*3*)	ERS213445	*pneumoniae*	K1257	ERR298797
9517_7#11	Pérez-Vázquez et al. (*3*)	ERS213446	*pneumoniae*	K1108	ERR298798
9517_7#12	Pérez-Vázquez et al. (*3*)	ERS213447	*pneumoniae*	K1286	ERR298799
9517_7#13	Pérez-Vázquez et al. (*3*)	ERS213448	*pneumoniae*	K1287	ERR298800
9517_7#14	Pérez-Vázquez et al. (*3*)	ERS213449	*pneumoniae*	K1367	ERR298801
9517_7#15	Pérez-Vázquez et al. (*3*)	ERS213450	*pneumoniae*	K1387	ERR298802
9517_7#16	Pérez-Vázquez et al (*3*)	ERS213451	*pneumoniae*	K1388	ERR298803
9517_7#17	Pérez-Vázquez et al. (*3*)	ERS213452	*pneumoniae*	K1363	ERR298804
9517_7#18	Pérez-Vázquez et al. (*3*)	ERS213453	*pneumoniae*	K1667	ERR298805
9517_7#19	Pérez-Vázquez et al. (*3*)	ERS213454	*pneumoniae*	K1668	ERR298806
9517_7#1	Pérez-Vázquez et al. (*3*)	ERS213436	*pneumoniae*	K830	ERR298788
9517_7#20	Pérez-Vázquez et al. (*3*)	ERS213455	*pneumoniae*	K1518	ERR298807
9517_7#21	Pérez-Vázquez et al. (*3*)	ERS213456	*pneumoniae*	K1579	ERR298808
9517_7#22	Pérez-Vázquez et al. (*3*)	ERS213457	*pneumoniae*	K1603	ERR298809
9517_7#23	Pérez-Vázquez et al. (*3*)	ERS213458	*pneumoniae*	K1539	ERR298810
9517_7#24	Pérez-Vázquez et al. (*3*)	ERS213459	*pneumoniae*	K1471	ERR298811
9517_7#25	Pérez-Vázquez et al. (*3*)	ERS213460	*pneumoniae*	K1641	ERR298812
9517_7#26	Pérez-Vázquez et al. (*3*)	ERS213461	*pneumoniae*	K1624	ERR298813

ID	Reference	Sample accession no.	Species	Strain	Accession no.
9517_7#27	Pérez-Vázquez et al. (*3*)	ERS213462	*pneumoniae*	K1623	ERR298814
9517_7#28	Pérez-Vázquez et al. (*3*)	ERS213463	*pneumoniae*	K1620	ERR298815
9517_7#2	Pérez-Vázquez et al. (*3*)	ERS213437	*pneumoniae*	K1075	ERR298789
9517_7#3	Pérez-Vázquez et al. (*3*)	ERS213438	*pneumoniae*	K1115	ERR298790
9517_7#4	Pérez-Vázquez et al. (*3*)	ERS213439	*pneumoniae*	K997	ERR298791
9517_7#5	Pérez-Vázquez et al. (*3*)	ERS213440	*pneumoniae*	K1004	ERR298792
9517_7#7	Pérez-Vázquez et al. (*3*)	ERS213442	*pneumoniae*	K1144	ERR298794
9517_7#8	Pérez-Vázquez et al. (*3*)	ERS213443	*pneumoniae*	K1148	ERR298795
9517_7#9	Pérez-Vázquez et al. (*3*)	ERS213444	*pneumoniae*	K1186	ERR298796
5235_2#4	Holt et al. (*2*)	ERS011989	*quasipneum*	QMP M1-977	ERR025611
5151_2#9	Holt et al. (*2*)	ERS011922	*quasipneum*	DR85/08	ERR025122
5193_3#1	Holt et al. (*2*)	ERS011830	*quasipneum*	K268An	ERR025488
5193_2#10	Holt et al. (*2*)	ERS011815	*quasipneum*	K38An	ERR025476
5197_2#12	Holt et al. (*2*)	ERS011829	*quasipneum*	K263An	ERR025556
5193_3#11	Holt et al. (*2*)	ERS011840	*quasipneum*	C-017-I-a-1	ERR025490
5193_5#4	Holt et al. (*2*)	ERS011845	*quasipneum*	033-CAZ-1	ERR025507
5193_5#6	Holt et al. (*2*)	ERS011847	*quasipneum*	073-CN-2	ERR025509
5193_6#1	Holt et al. (*2*)	ERS011854	*quasipneum*	EW-60-R-MAG-2	ERR025514
5197_2#5	Holt et al. (*2*)	ERS011822	*quasipneum*	K222Ca	ERR025560
5197_2#4	Holt et al. (*2*)	ERS011821	*quasipneum*	K215Ax	ERR025559
5193_2#3	Holt et al. (*2*)	ERS011808	*quasipneum*	NCSR130	ERR025480
5193_2#4	Holt et al. (*2*)	ERS011809	*quasipneum*	BAL073	ERR025481
5193_8#9	Holt et al. (*2*)	ERS011910	*quasipneum*	DU35427/05	ERR025551
5150_2#1	Holt et al. (*2*)	ERS005752	*quasipneum*	AJ055	ERR024830
5193_7#10	Holt et al. (*2*)	ERS011887	*quasipneum*	UI 4256	ERR025528
5193_7#8	Holt et al. (*2*)	ERS011885	*quasipneum*	UI 2877	ERR025537
5197_8#5	Holt et al. (*2*)	ERS011894	*quasipneum*	UI 9552	ERR025586
5197_8#3	Holt et al. (*2*)	ERS011892	*quasipneum*	UI 7631	ERR025584
5151_2#12	Holt et al. (*2*)	ERS011925	*variicola*	QMP B2-481	ERR025114
5193_8#7	Holt et al. (*2*)	ERS011908	*variicola*	QMP B2-288	ERR025549
5151_2#7	Holt et al. (*2*)	ERS011920	*variicola*	QMP B2-340	ERR025120
5151_3#1	Holt et al. (*2*)	ERS011926	*variicola*	QMP B2-483	ERR025124
5151_5#10	Holt et al. (*2*)	ERS011947	*variicola*	QMP M1-765	ERR025138
5151_5#9	Holt et al. (*2*)	ERS011946	*variicola*	QMP M1-763	ERR025148
5151_6#12	Holt et al. (*2*)	ERS011961	*variicola*	QMP M1-428	ERR025153
5151_5#6	Holt et al. (*2*)	ERS011943	*variicola*	QMP M1-726	ERR025145
5299_1#12	Holt et al. (*2*)	ERS011973	*variicola*	QMP M1-888	ERR025982
5235_1#4	Holt et al. (*2*)	ERS011977	*variicola*	QMP M1-893	ERR025598
5197_7#5	Holt et al. (*2*)	ERS011870	*variicola*	Pus 13542	ERR025573
5235_8#10	Holt et al. (*2*)	ERS011792	*variicola*	08-109P	ERR025671
5193_1#10	Holt et al. (*2*)	ERS011803	*variicola*	1892m	ERR025463
5150_1#7	Holt et al. (*2*)	ERS005747	*variicola*	AJ026	ERR024826
5150_3#8	Holt et al. (*2*)	ERS005771	*variicola*	AJ182	ERR024853
5150_1#8	Holt et al. (*2*)	ERS005748	*variicola*	AJ027	ERR024827
5150_2#2	Holt et al. (*2*)	ERS005754	*variicola*	AJ135	ERR024834
5150_5#2	Holt et al. (*2*)	ERS005778	*variicola*	AJ292	ERR025102
5235_5#1	Holt et al. (*2*)	ERS012010	*quasipneum*	QMP Z4-726	ERR025631

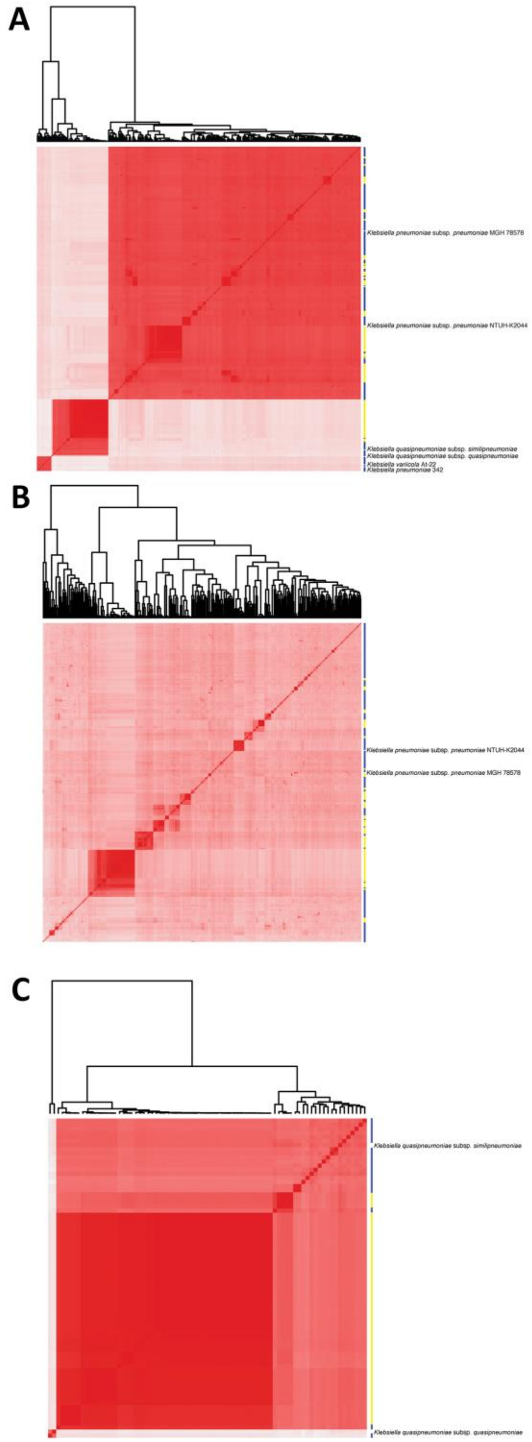

Technical Appendix Figure 1. Whole-genome clustering. Cluster analysis using mash (*4*) for the rapid identification of species was used to identify putative members of KpI (*Klebsiella pneumoniae*), KpII (*K. quasipneumoniae*) and KpIII (*K. variicola*) by comparison of several reference strains and the global collection ([*2*] blue bar) with the isolates from this study (yellow bar). In-depth analysis indicates a large

diversity within KpI (B) and a large group of highly similar members of KpII (C), most similar to the reference *K. quasipneumoniae subsp. similipneumoniae* (in other literature also referred to as KpIIA).

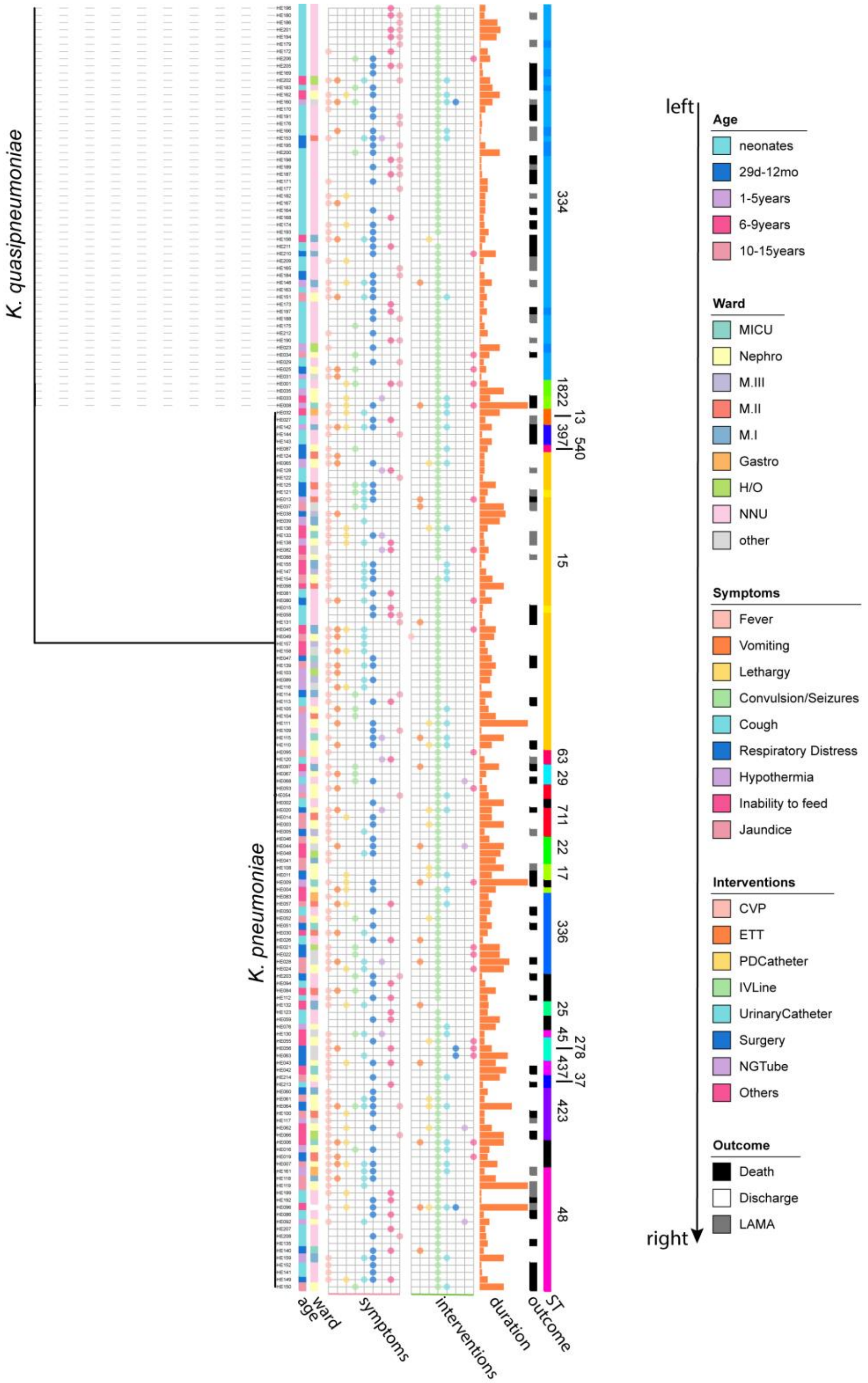

Technical Appendix Figure 2. Patient metadata in phylogenetic context. The guidance tree is based on the core gene alignment for this strain set generated by roary. The metadata are shown as depicted in the legends, Intravenous lines (cannulas) were used to administer dextrose saline, mannitol and antimicrobial drugs if oral treatment was not possible. STs with <3 members are shown in black; shading within a

sequence type (e.g., bright and dark yellow for ST15) indicates uncertain predictions (e.g., ST15~). CVP, central venous pressure (line); ETT, endotracheal tube; gastro, gastroenterology; H/O, hematology oncology; IV, intravenous; LAMA, left against medical advice; M, medical unit; MICU, medical intensive care unit; NG, nasogastric; NNU, neonatal nursery; ST, sequence type.

References

1. Chung The H, Karkey A, Pham Thanh D, Boinett CJ, Cain AK, Ellington M, et al. A high-resolution genomic analysis of multidrug-resistant hospital outbreaks of *Klebsiella pneumoniae*. EMBO Mol Med. 2015;7:227–39. PubMed http://dx.doi.org/10.15252/emmm.201404767

2. Holt KE, Wertheim H, Zadoks RN, Baker S, Whitehouse CA, Dance D, et al. Genomic analysis of diversity, population structure, virulence, and antimicrobial resistance in *Klebsiella pneumoniae*, an urgent threat to public health. Proc Natl Acad Sci U S A. 2015;112:E3574–81. PubMed http://dx.doi.org/10.1073/pnas.1501049112

3. Pérez-Vázquez M, Oteo J, García-Cobos S, Aracil B, Harris SR, Ortega A, et al. Phylogeny, resistome and mobile genetic elements of emergent OXA-48 and OXA-245 *Klebsiella pneumoniae* clones circulating in Spain. J Antimicrob Chemother. 2016;71:887–96. PubMed http://dx.doi.org/10.1093/jac/dkv458

4. Ondov BD, Treangen TJ, Melsted P, Mallonee AB, Bergman NH, Koren S, et al. Mash: fast genome and metagenome distance estimation using MinHash. Genome Biol. 2016;17:132. PubMed http://dx.doi.org/10.1186/s13059-016-0997-x
